# Disproportionality analysis of taste disorders using the FDA adverse event reporting system and Japanese adverse drug event report databases

**DOI:** 10.3389/fphar.2025.1659746

**Published:** 2025-11-27

**Authors:** Yoji Kyotani, Kiichi Nakahira, Masanori Yoshizumi

**Affiliations:** Department of Pharmacology, Nara Medical University School of Medicine, Nara, Japan

**Keywords:** taste disorder, FDA adverse event reporting system (FAERS), Japanese AdverseDrug event report (JADER), drug efficacy, dysgeusia, adverse event

## Abstract

**Introduction:**

Taste disorders, which can diminish the quality of life and potentially affect drug treatment efficacy, are adverse events that may occur with the use of various medications; however, only a few studies have comprehensively examined this relationship. Spontaneous reporting system-based databases, such as the FDA Adverse Event Reporting System (FAERS) and Japanese Adverse Drug Event Report (JADER), are efficient sources of information for patients treated with drugs; however, analyses using this type of database have some limitations. To address these gaps in knowledge, we comprehensively analyzed the association between drugs and taste disorders using the FAERS and JADER databases.

**Methods:**

The reporting odds ratio (ROR) and Bayesian statistics (Bayesian Confidence Propagation Neural Network, BCPNN) from these databases were used to determine the association between drugs and taste disorders. Weibull distribution and logistic regression were used to investigate the time-to-onset characteristics and odds ratios adjusted for age and sex, respectively, for taste disorders, using data from the FAERS database.

**Results and discussion:**

Of the 130 drugs for which a signal was detected in both ROR and BCPNN in the FAERS database, 14 had similar signals in the JADER database. Onset time and logistic regression analyses were performed using the FAERS database to characterize drug-induced taste disorders. The median onset time was the same day of administration for nirmatrelvir/ritonavir and clarithromycin; otherwise, it varied among the other drugs. The hazard for clarithromycin remained constant throughout administration, while the hazards for the other drugs decreased over time. Logistic regression analysis confirmed significant associations between the drugs and taste disorders, even after adjusting for age and sex. Overall, these results indicate that certain drugs may be involved in taste disorders, which, depending on their characteristics, warrants further caution and attention. The findings of this study may help healthcare providers recognize drug-induced taste disorders that can lead to a reduced quality of life and noncompliance with medication.

## 1 Introduction

Taste disorders, or dysgeusia, do not typically result in death. However, they can diminish patients’ quality of life by reducing their satisfaction with food, decreasing food intake, and increasing health risks. Moreover, an impaired capacity to differentiate between varying intensities or concentrations of tastes may lead to excess intake of salt and sugar, resulting in the development of chronic diseases, including diabetes mellitus and heart failure (Syed et al., 2016; Jafari et al., 2021). Taste disorders have been frequently analyzed in conjunction with olfactory disorders (Kan et al., 2021; Hamazaki and Uesawa, 2024). However, olfactory disorders have no reasonable influence on taste disorders, and the clinical association between smell and taste dysfunction may reflect comorbid influences (Stinton et al., 2010). Comorbid smell and taste dysfunctions are much less common than either one (Liu et al., 2016). The National Health and Nutrition Examination Survey (NHANES 2013–2014) previously reported the estimated prevalence of taste impairment in the United States population aged 40 years and older at 17.3% (Liu et al., 2016). Limited to adverse events during pharmacotherapy, Shinkai et al. reported 33% taste alterations in the Oral Health: San Antonio Longitudinal Study of Aging (OH:SALSA) (Shinkai et al., 2006). In contrast, the incidence of drug-induced taste disorders in Japan is approximately 17% (Nin et al., 2017). Drug-related taste disorders may be caused by various medications (Naik et al., 2010; Rademacher et al., 2020; Tuccori et al., 2011; Syed et al., 2016); however, the findings among these studies are not necessarily consistent. The identification of adverse events that were undetected prior to marketing relies on post-marketing surveillance and spontaneous reporting systems (SRSs) because clinical research focused on drug-induced adverse events is ethically unacceptable. However, few studies have specifically examined taste disorders using SRS. Most of these studies relied on a single SRS, and the results have not been validated by other SRSs employing the same methodology. Consequently, there is a paucity of studies that have conducted comprehensive analyses of taste disorders using multiple SRSs.

The Food and Drug Administration Adverse Event Reporting System (FAERS) is a passive surveillance database freely provided by the Food and Drug Administration (FDA) of the United States (CIOMS Working Group VIII, 2010). A similar database, the Japanese Adverse Drug Event Report (JADER), is an early post-marketing phase vigilance database freely provided by the Pharmaceuticals and Medical Devices Agency (PMDA) of Japan (CIOMS Working Group VIII, 2010). These databases are large-scale SRSs focusing on post-marketing safety surveillance (Kyotani et al., 2023a; 2023b). SRSs have several limitations, including reporting bias, underreporting of adverse events (AEs), and variability in reporting quality. However, they are useful for detecting signals, indicating a possible causal relationship between an AE and a drug. Additionally, confirming the signals using another database is advantageous because it complements some of the limitations and strengthens the reliability of the results.

This study aimed to identify drugs associated with taste disorders by comprehensively analyzing FAERS and JADER data and using the FAERS database to evaluate the time-to-onset characteristics and age- and sex-adjusted odds ratios for drugs whose signals were identified in both databases. The results of this study may assist healthcare providers in recognizing drug-induced taste disorders and help patients who experience reduced quality of life and noncompliance with medication due to these disorders.

## 2 Materials and methods

### 2.1 Data sources

FAERS data from Q1 2015 to Q4 2024 were obtained from the FDA website (www.fda.gov). The FAERS dataset included seven tables: DEMO, DRUG, REAC, OUTC, RPSR, THER, and INDI. For the FAERS data, OpenRefine was used for character encoding and TSV file conversion and was utilized to efficiently incorporate data into the database. In the present study, we combined data from the DEMO, DRUG, and REAC tables and limited the analysis to primary suspect drugs (PS), considering the drug’s role in the event. As outlined in the FAERS Public Dashboard, each unique case submission is assigned a version number in the FAERS. If a follow-up is received for a previously submitted case, the next number is assigned to that version. Therefore, the latest version of each case was extracted in the present study to eliminate duplicates. Blank or anomalous data regarding age, sex, start date of administration, and event date of AE occurrence were excluded. For the time-to-onset analysis, the data were modified as follows: (1) records with only the year of treatment onset or AEs were excluded; (2) data with the year and month were set to 15 days; (3) data with the taste disorder onset date before the start of treatment were excluded; and (4) the period was set to 0.5 days for data in which the onset date of taste disorders was the same as the start date of drug administration.

Data from the JADER database between April 2004 and October 2024 were obtained from the PMDA website (www.pmda.go.jp; available only in Japanese). The structure of the JADER database adheres to international safety reporting guidelines (ICH E2B). The JADER dataset included four tables: patient demographic information (DEMO), drug information (DRUG), AEs (REAC), and primary diseases (HIST). The JADER data were disorganized and required extensive cleaning before it could be integrated into the database. To address this, we utilized the Text Editor and OpenRefine for several tasks, including merging records with obvious input errors, processing fields containing multiple entries, removing the time portion from date/year entries that included time, converting data marked as “unknown” to NULL values, and correcting inconsistencies between full-width and half-width characters. The DEMO table was linked to the DRUG and REAC tables using ID numbers. Blank and unknown data regarding sex, age, start date of administration, event date of AE occurrence, and duplicate entries were excluded. Drugs with a role code of “suspected drug” were included. The date processing used in FAERS has been applied to the JADER database.

### 2.2 Definition of adverse events and drug names

According to the terminology preferred by the Medical Dictionary for Regulatory Activities version 27.0 (MedDRA v27.0), case reports on taste disorders were extracted using the following terms: taste disorder [preferred term (PT) code: 10082490], dysgeusia (PT code: 10013911), hypogeusia (PT code: 10020989), and ageusia (PT code: 10001480) (Su et al., 2013; Nin and Tsuzuki, 2024). Drug names were referred to using international non-proprietary names (INN) or United States Adopted Names (USAN). For the JADAR database, drugs not described by INN or USAN were referred to using the Japanese Pharmacopoeia, 18th edition (JP18).

### 2.3 Signal detection

A 2 × 2 contingency table was created (Table 1) and used to detect signals using reporting odds ratios (RORs) and Bayesian Confidence Propagation Neural Network (BCPNN). RORs and BCPNN were examined for drugs reported in at least five cases of target AEs.

**TABLE 1:** Representative 2 × 2 contingency table used in this study.

	Target AE	All other AEs	Total
Target drug	N_11_	N_10_	N_1+_
All other drugs	N_01_	N_00_	N_0+_
Total	N_+1_	N_+0_	N_++_

AE, adverse event.

#### 2.3.1 ROR

ROR was used as a signal index in the Netherlands Pharmacovigilance Centre Lareb and is recommended as a signal detection method by the European Medicines Agency (EMA) (CIOMS Working Group VIII, 2010; European Medicines Agency, 2016). The ROR and 95% confidence intervals (CIs) of the AEs related to the target drug were calculated, as shown in Table 1 and Equations 1 and 2:

$$ROR=\frac{N_{11}/N_{10}}{N_{01}/N_{00}}$$ (1)

$$ROR\left(95\%\;CI\right)=e^{\ln\left(ROR\right)\pm 1.96\sqrt{\frac{1}{N_{11}}+\frac{1}{N_{10}}+\frac{1}{N_{01}}+\frac{1}{N_{00}}}}$$ (2)

A signal was defined as the lower bound of the 95% CI for ROR >1.

#### 2.3.2 BCPNN

BCPNN is a signal detection index used by the World Health Organization (CIOMS Working Group VIII, 2010). The signal was evaluated using the information component (IC) of the BCPNN, a method based on the Bayesian statistics model (Bate et al., 1998; Szarfman et al., 2002; Noguchi et al., 2021). IC and the lower end of the 95% credible interval for IC (IC_025_) were calculated using Equations 3–6.

$$E\left({IC}_{11}\right)=\log_{2}\frac{\left(N_{11}+\gamma_{11}\right)\left(N_{++}+\alpha \right)\left(N_{++}+\beta \right)}{\left(N_{++}+\gamma \right)\left(N_{1+}+\alpha_{1}\right)\left(N_{+1}+\beta_{1}\right)}$$ (3)

$$V\left({IC}_{11}\right)={\left(\frac{1}{\log2}\right)}^{2}\left[\frac{N_{++}-N_{11}+\gamma -\gamma_{11}}{\left(N_{11}+\gamma_{11}\right)\left(1+N_{++}+\gamma \right)}+\frac{N_{++}-N_{1+}+\alpha -\alpha_{1}}{\left(N_{1+}+\alpha_{1}\right)\left(1+N_{++}+\alpha \right)}+\frac{N_{++}-N_{+1}+\beta -\beta_{1}}{\left(N_{+1}+\beta_{1}\right)\left(1+N_{++}+\beta \right)}\right.]$$ (4)

$$\gamma =\gamma_{11}\frac{\left(N_{++}+\alpha \right)\left(N_{++}+\beta \right)}{\left(N_{1+}+\alpha_{1}\right)\left(N_{+1}+\beta_{1}\right)},\gamma_{11}=1,\alpha_{1}=\beta_{1}=1,\alpha =\beta =2$$ (5)

$$IC\;\left(95\%\;credible\;interval\right)=E\left({IC}_{11}\pm 2\sqrt{V\left({IC}_{11}\right)}\right)$$ (6)

A signal was defined when the IC_025_ was >0.

### 2.4 Time-to-onset analysis

The Weibull distribution is a continuous probability distribution describing the nature of the time to failure and includes a scale parameter and a shape parameter (β). In SRS data analysis, the shape parameter β, which determines the shape of the distribution function, was used to indicate the hazard without a reference population (Abe et al., 2016; Nakao et al., 2017; Yamashiro et al., 2022; Kyotani et al., 2023a). Therefore, in this study, the parameters were calculated using a Weibull plot. For β = 1, the hazard was estimated to be constant over time (random failure). If the lower bound of the 95% CI of β was >1, the hazard was considered to increase over time (wear-out failure). Finally, if the upper bound of the 95% CI of β was <1, the hazard decreased over time (early failure). In the final analysis, the subjects were limited to drugs that were implicated in a minimum of 20 cases of taste disorders, and the cumulative distribution function was obtained using the mean rank if the sample size was ≥30 and the median rank if the sample size was <30.

### 2.5 Logistic regression analysis

Logistic regression analysis is often used to account for covariables. Age and sex were assumed to be variables and included as explanatory variables. The number of events per variable (EPV) was set to 10. Therefore, the adjusted RORs were examined for drugs reported in at least 30 cases of the target AE as follows:

$$\log\left(odds\right)=\beta_{0}+\beta_{1}D+\beta_{1}A+\beta_{2}G$$ (7)

where D, A, and G indicate drug, age, and sex, respectively (Equation 7). There was no multicollinearity, as all values of the variance inflation factor (VIF) were <2.

### 2.6 Statistical analysis

All statistical analyses were conducted using Python (version 3.12) and the statsmodels package (version 0.14.4). Some results from each analysis were verified using Microsoft Excel 2021 (version 2507) or R software (version 4.4.2).

## 3 Results

### 3.1 Characteristics of patients with taste disorders in the FAERS and JADER databases

**FIGURE 1:**
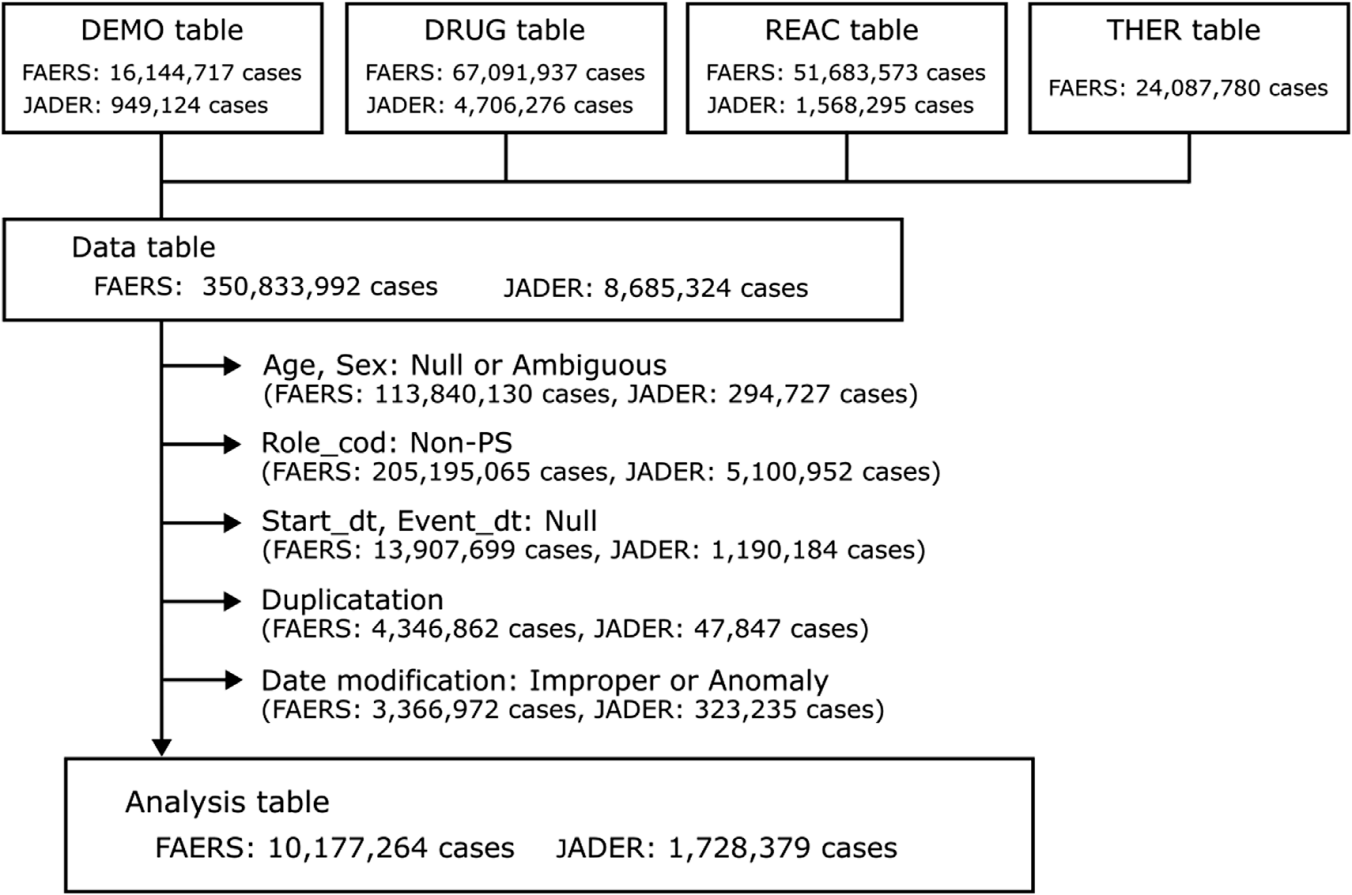
Flowchart of data construction.

During the study period, the FAERS and JADER databases received 16,144,717 reports, while the JADER database received 949,124 reports. Following the implementation of appropriate procedures for duplicate data and missing or ambiguous data for age, sex, initial date of administration, and event date, 10,177,264 and 1,728,379 cases from the FAERS and JADER databases, respectively, were used in the present study (Figure 1). Of these, 24,485 and 1,140 cases were identified as taste disorders in the FAERS and JADER databases, respectively. The characteristics of the taste disorder data are presented in Table 2.

**TABLE 2:** Demographic characteristics of taste disorders reported in the FAERS and JADER databases.

Characteristics	FAERS Case number (%)	JADER Case number (%)
Adverse event		
Taste disorder	4,168 (17.0)	662 (58.1)
Dysgeusia	15,366 (62.8)	344 (30.2)
Ageusia	4,581 (18.7)	97 (8.5)
Hypogeusia	370 (1.5)	37 (3.2)
Sex		
Male	8,628 (35.2)	584 (51.2)
Female	15,857 (64.8)	556 (48.8)
Age (years)		
<19	312 (1.3)	19 (1.7)
20–29	1,205 (4.9)	12 (1.1)
30–39	2,016 (8.2)	72 (6.3)
40–49	2,834 (11.6)	114 (10.0)
50–59	4,609 (18.8)	252 (22.1)
60–69	6,343 (25.9)	312 (27.4)
70–79	5,309 (21.7)	257 (22.5)
80–89	1,700 (6.9)	95 (8.3)
>90	157 (0.6)	7 (0.6)
Occupation of reporter		
Consumer	14,150 (57.8)	10 (0.9)
Other health-professional	4,338(17.7)	26 (2.3)
Physician	3,188 (13.0)	962 (84.4)
Pharmacist	2,078 (8.5)	133 (11.7)
Lawer	26 (0.1)	0 (0.0)
Missing	705 (2.9)	9 (0.8)

### 3.2 Signal detection

Signals were detected using ROR and BCPNN, as well as the FAERS and JADER databases, to ensure more reliable results. A total of 143 drugs from the FAERS database exhibited both ROR and BCPNN signals related to taste disorders (Supplementary Table S1). Similar analyses were conducted with the JADER database to validate the results. Signals in both ROR and BCPNN were identified for 31 drugs in the JADER database (Supplementary Table S2). The following medications showed signals in both the FAERS and JADER databases: nirmatrelvir/ritonavir (FAERS ROR = 27.04, IC = 4.43; JADER ROR = 24.96, IC = 3.12), sunitinib malate (FAERS ROR = 7.59, IC = 2.86; JADER ROR = 3.65, IC = 1.70), clarithromycin (FAERS ROR = 12.18, IC = 3.51; JADER ROR = 7.75, IC = 2.45), enzalutamide (FAERS ROR = 2.90, IC = 1.51; JADER ROR = 7.90, IC = 2.27), palbociclib (FAERS ROR = 1.60, IC = 0.66; JADER ROR = 3.12, IC = 1.20), pazopanib hydrochloride (FAERS ROR = 3.51, IC = 1.77; JADER ROR = 3.10, IC = 1.19), terbinafine hydrochloride (FAERS ROR = 26.32, IC = 4.33; JADER ROR = 8.89, IC = 2.30), crizotinib (FAERS ROR = 3.63, IC = 1.80; JADER ROR = 11.31, IC = 2.80), fluorouracil (FAERS ROR = 1.84, IC = 0.86; JADER ROR = 1.66, IC = 0.68), varenicline tartrate (FAERS ROR = 2.31, IC = 1.17; JADER ROR = 11.29, IC = 2.76), enfortumab vedotin (FAERS ROR = 8.17, IC = 2.77; JADER ROR = 15.61, IC = 3.25), panitumumab (FAERS ROR = 1.82, IC = 0.83; JADER ROR = 3.31, IC = 1.35), romidepsin (FAERS ROR = 3.64, IC = 1.55; JADER ROR = 13.86, IC = 2.13), and vorinostat (FAERS ROR = 6.03, IC = 1.94; JADER ROR = 47.9, IC = 4.22) (Table 3). Of the 14 drugs for which signals were detected in both databases, 10 were classified as “antineoplastic and immunomodulating agents (L)” according to the Anatomical Therapeutic Chemical (ATC) classification system. This was followed by two “antiinfective for systemic use (J),” one as “dermatologicals (D),” and one as “nervous system.”

**TABLE 3:** RORs and ICs of drugs associated with taste disorders in both the FAERS and JADER databases.

Drug	FEARS					
	ATC code	Cases	Non-cases	Total	ROR (95% CI)	IC (IC025)
Total		24,485	10,152,779	10,177,264		
Nirmatrelvir/ritonavir	J05AE30	3,968	72,107	76,075	27.04 (26.11–27.99)	4.43 (4.38)
Sunitinib malate	L01XJ01	514	28,585	29,099	7.59 (6.95–8.29)	2.86 (2.73)
Clarithromycin	J01FA09	419	14,487	14,906	12.18 (11.05–13.44)	3.51 (3.37)
Enzalutamide	L02BB04	358	51,749	52,107	2.90 (2.61–3.22)	1.51 (1.35)
Palbociclib	L01EF01	259	67,521	67,780	1.60 (1.41–1.81)	0.66 (0.48)
Pazopanib hydrochloride	L01EX03	155	18,404	18,559	3.51 (2.99–4.11)	1.77 (1.54)
Terbinafine hydrochloride	D01AE15	104	1,645	1,749	26.32 (21.58–32.10)	4.33 (4.04)
Crizotinib	L01ED01	76	8,697	8,773	3.63 (2.90–4.55)	1.80 (1.47)
Fluorouracil	L01BC02	57	12,833	12,890	1.84 (1.42–2.39)	0.86 (0.48)
Varenicline tartrate	N07BA03	52	9,353	9,405	2.31 (1.76–3.03)	1.17 (0.77)
Enfortumab vedotin	L01FX13	39	1,983	2,022	8.17 (5.95–11.21)	2.77 (2.31)
Panitumumab	L01FE02	33	7,525	7,558	1.82 (1.29–2.56)	0.83 (0.33)
Romidepsin	L01XH02	10	1,139	1,149	3.64 (1.95–6.79)	1.55 (0.67)
Vorinostat	L01XH01	8	550	558	6.03 (3.00–12.13)	1.94 (0.97)

Drug	JADER					
	ATC code	Cases	Non-cases	Total	ROR (95% CI)	IC (CI025)
Total		1,140	1,727,239	1,728,379		
Vorinostat	L01XH01	30	974	1,004	47.90 (33.17–69.21)	4.22 (3.69)
Fluorouracil	L01BC02	27	24,905	24,932	1.66 (1.13–2.43)	0.68 (0.13)
Enfortumab vedotin	L01FX13	23	2,276	2,299	15.61 (10.31–23.63)	3.25 (2.65)
Sunitinib malate	L01XJ01	23	9,685	9,708	3.65 (2.42–5.52)	1.70 (1.10)
Crizotinib	L01ED01	16	2,172	2,188	11.31 (6.89–18.55)	2.80 (2.09)
Clarithromycin	J01FA09	16	3,167	3,183	7.75 (4.73–12.71)	2.45 (1.75)
Varenicline tartrate	N07BA03	15	2,038	2,053	11.29 (6.77–18.82)	2.76 (2.03)
Nirmatrelvir/ritonavir	J05AE30	12	736	748	24.96 (14.07–44.28)	3.12 (2.31)
Enzalutamide	L02BB04	10	1,932	1,942	7.90 (4.23–14.75)	2.27 (1.39)
Terbinafine hydrochloride	D01AE15	9	1,545	1,554	8.89 (4.60–17.16)	2.30 (1.38)
Panitumumab	L01FE02	7	3,220	3,227	3.31 (1.57–6.96)	1.35 (0.33)
Romidepsin	L01XH02	5	549	554	13.86 (5.73–33.49)	2.13 (0.95)
Palbociclib	L01EF01	5	2,437	2,442	3.12 (1.29–7.51)	1.20 (0.02)
Pazopanib hydrochloride	L01EX03	5	2,452	2,457	3.10 (1.29–7.47)	1.19 (0.01)

ATC, anatomical therapeutic chemical; ROR, reporting odds ratio; IC, information component; CI, confidence interval.

### 3.3 Onset time and failure pattern of taste disorders

The Weibull distribution was used to analyze the onset time and failure patterns of taste disorders using the FAERS database (Supplementary Table S3). However, it is important to note that the reliability of the results may not be high, as the assumption of the Weibull distribution is that taste disorders develop consistently over an infinite time period. The results of the time-to-onset analysis for the signal-detected drugs in both the FAERS and JADER databases are presented in Table 4. Nirmatrelvir/ritonavir (β = 0.88, median = 0.5), sunitinib malate (β = 0.53, median = 16.5), clarithromycin (β = 1.07, median = 0.5), enzalutamide (β = 0.56, median = 54.5), palbociclib (β = 0.51, median = 29.0), pazopanib hydrochloride (β = 0.55, median = 18.0), terbinafine hydrochloride (β = 0.86, median = 30), crizotinib (β = 0.50, median = 9.5), fluorouracil (β = 0.71, median = 11), varenicline tartrate (β = 0.52, median = 6.0), enfortumab vedotin (β = 0.89, median = 14.0), and panitumumab (β = 0.60, median = 49) were predicted to exhibit early failure patterns; thus, the hazards of these drugs decreased over time. Only clarithromycin was expected to show random failure; therefore, the drug hazard was considered constant over time. No drugs were estimated to cause wear-out failure. Additionally, the median time-to-onset varied depending on the drug. The median values for nirmatrelvir/ritonavir, clarithromycin, and varenicline tartrate were less than 1 week, with the median values for the first two drugs occurring on the day of medication. The median values for enzalutamide, palbociclib, terbinafine hydrochloride, and panitumumab exceeded 4 weeks.

**TABLE 4:** Shape parameter β of the Weibull distribution and the failure pattern for each drug associated with taste disorders in the FAERS database.

Drug	Cases	Mean time	Time-to-onset (IQR)	Shape parameter β	Pattern
Nirmatrelvir/ritonavir	3,968	2.0	0.5 (0.5, 1.0)	0.88 (0.85–0.91)	Early failure
Sunitinib malate	514	107.9	16.5 (4.0, 61.0)	0.53 (0.51–0.55)	Early failure
Clarithromycin	419	1.4	0.5 (0.5, 1.0)	1.07 (0.97–1.18)	Random failure
Enzalutamide	358	114.3	54.5 (11.5, 160.0)	0.56 (0.54–0.58)	Early failure
Palbociclib	259	131.3	29.0 (6.0, 150.5)	0.51 (0.49–0.53)	Early failure
Pazopanib hydrochloride	155	81.3	18.0 (2.5, 85.0)	0.55 (0.52–0.58)	Early failure
Terbinafine hydrochloride	104	36.0	30.0 (18.5, 46.0)	0.86 (0.80–0.91)	Early failure
Crizotinib	76	111.6	9.5 (2.0, 59.0)	0.50 (0.46–0.55)	Early failure
Fluorouracil	57	23.4	11.0 (3.0, 30.0)	0.71 (0.66–0.76)	Early failure
Varenicline tartrate	52	43.7	6.0 (0.5, 17.0)	0.52 (0.45–0.60)	Early failure
Enfortumab vedotin	39	22.5	14.0 (7.0, 20.5)	0.89 (0.81–0.97)	Early failure
Panitumumab	33	88.8	49.0 (12.0, 112.0)	0.60 (0.57–0.63)	Early failure

### 3.4 Age- and sex-adjusted RORs

Sex and age are typically considered variables in safety data analysis. Therefore, in this study, we aimed to evaluate the association between drugs and taste disorders, accounting for the effects of these variables. The adjusted ROR was calculated using logistic regression analysis to account for the impact of patient age and sex on the FAERS database (Supplementary Table S4). However, since the EPV was set to 10, only drugs with at least 30 reports of taste disorders were included. The results of the study on signal-detected drugs in both the FAERS and JADER databases are presented in Table 5. Significant associations were identified for the following drugs: nirmatrelvir/ritonavir (adjusted ROR = 26.02), sunitinib malate (adjusted ROR = 7.25), clarithromycin (adjusted ROR = 12.66), enzalutamide (adjusted ROR = 2.62), palbociclib (adjusted ROR = 1.37), pazopanib hydrochloride (adjusted ROR = 3.39), terbinafine hydrochloride (adjusted ROR = 26.28), crizotinib (adjusted ROR = 3.47), fluorouracil (adjusted ROR = 1.73), varenicline tartrate (adjusted ROR = 2.36), enfortumab vedotin (adjusted ROR = 7.46), and panitumumab (adjusted ROR = 1.74).

**TABLE 5:** ROR of taste disorders adjusted for age and sex in the FAERS database.

Drug	Cases	Total	Crude ROR (95% CI)	Adjusted ROR (95% CI)
Nirmatrelvir/ritonavir	3,968	76,075	27.04 (26.11–27.99)	26.02 (25.12–26.94)
Sunitinib malate	514	29,099	7.59 (6.95–8.29)	7.25 (6.64–7.92)
Clarithromycin	419	14,906	12.18 (11.05–13.44)	12.66 (11.47–13.96)
Enzalutamide	358	52,107	2.90 (2.61–3.22)	2.62 (2.36–2.92)
Palbociclib	259	67,780	1.60 (1.41–1.81)	1.37 (1.21–1.55)
Pazopanib hydrochloride	155	18,559	3.51 (2.99–4.11)	3.39 (2.89–3.97)
Terbinafine hydrochloride	104	1,749	26.32 (21.58–32.10)	26.28 (21.54–32.06)
Crizotinib	76	8,773	3.63 (2.90–4.55)	3.47 (2.77–4.35)
Fluorouracil	57	12,890	1.84 (1.42–2.39)	1.73 (1.33–2.25)
Varenicline tartrate	52	9,405	2.31 (1.76–3.03)	2.36 (1.80–3.10)
Enfortumab vedotin	39	2,022	8.17 (5.95–11.21)	7.46 (5.43–10.24)
Panitumumab	33	7,558	1.82 (1.29–2.56)	1.74 (1.24–2.45)

ROR, reporting odds ratio; CI, confidence interval.

## 4 Discussion

Taste disorders, affecting the patients’ quality of life, are AEs that may occur with the use of various medications. However, only a few studies have comprehensively examined this relationship. Pharmacovigilance studies using SRS are useful for identifying AEs that have not been detected in clinical trials and are used in many countries (CIOMS Working Group VIII, 2010; European Medicines Agency, 2016). In the present study, the FAERS and JADER databases were used to identify signals between drugs and taste disorders. Furthermore, the onset time characteristics and adjusted ROR by age and sex for these drugs were estimated using the FAERS database. We inferred the following from our results: (1) 14 drugs were associated with taste disorders in both the FAERS and JADER databases; (2) the median onset of taste disorders for nirmatrelvir/ritonavir and clarithromycin occurred on the day of administration; (3) the failure pattern of taste disorders remained consistent over time for clarithromycin but decreased over time for the other drugs; and (4) 12 drugs that had at least 30 cases of taste disorder and exhibited significant signals in both the FAERS and JADER databases were significantly associated with taste disorders regardless of age and sex.

Signal detection in AE-reporting systems is a critical step in risk management. It is often performed using ROR and BCPNN, which are established methods for detecting adverse drug reactions (Bate et al., 1998; Szarfman et al., 2002; van Puijenbroek et al., 2002; Noguchi et al., 2021; CIOMS Working Group VIII, 2010). In the present study, the ROR and BCPNN were used for signal detection to evaluate the association of drugs with taste disorders. They revealed significant associations for 14 drugs in both the FAERS and JADER databases: nirmatrelvir/ritonavir, sunitinib malate, clarithromycin, enzalutamide, palbociclib, pazopanib hydrochloride, terbinafine hydrochloride, crizotinib, fluorouracil, varenicline tartrate, enfortumab vedotin, panitumumab, romidepsin, and vorinostat (Table 3). With the exception of palbociclib and vorinostat, these drugs have been reported to be associated with taste disorders (Han and Youker, 2011; Tuccori et al., 2011; Cooper et al., 2013; Imai et al., 2013; Bayraktar-Ekincioglu and Kucuk, 2018; Kawahara et al., 2019; Ueno et al., 2019; Van Elst et al., 2022; Akhvlediani et al., 2023; Fu et al., 2025; Miyake et al., 2025). The results of this study support the findings of these previous studies. However, most of them were either limited to a single database or case report or inferred AEs that included both taste and smell disturbances. Vorinostat, romidepsin, enfortumab vedotin, and terbinafine hydrochloride had counts with expected frequencies of less than five in the FAERS. Although expected frequency is not directly included in the signal criteria of the ROR or BCPNN, it is important to note that the expected frequency is low. This is because the proportional reporting rate (PRR), another signal detection method, uses the chi-square test as part of its criteria, and the results are similar to those of ROR. Our findings in the present study provide valuable evidence for drugs associated with taste disorders.

Drug-induced taste disorders are a significant form of taste disorders. They are thought to be caused by various factors, including cytotoxic effects, zinc and copper deficiency, purinergic receptor antagonism, and calcium inflow blockade (Naik et al., 2010; Yagi et al., 2013; Jafari et al., 2021; Nin and Tsuzuki, 2024). Nirmatrelvir/ritonavir, the most frequently reported taste disorder in the FAERS database, is used to treat COVID-19 and can cause dysgeusia associated with the bitter taste receptor TAS2Rs (Akhvlediani et al., 2023; Brooks et al., 2023). Among the drugs showing signals in the FAERS and JADER databases, clarithromycin has been used for brief periods to treat infections. Although clarithromycin is associated with taste disorders, the underlying mechanisms remain unclear (Tuccori et al., 2011). The time to the onset of taste disorders for these drugs, briefly used to treat infections, was primarily from the day of administration to the next day (Table 4). Considering the failure pattern of these drugs, nirmatrelvir/ritonavir can cause taste disorders from the day after administration, and the risk is highest during this period. The risk of taste disorders with erythromycin remained constant throughout the administration period, despite the shorter duration of administration.

The drugs most commonly associated with taste disorders were antineoplastic and immunomodulating agents, including sunitinib malate, enzalutamide, palbociclib, pazopanib hydrochloride, crizotinib, fluorouracil, enfortumab vedotin, panitumumab, romidepsin, and vorinostat, according to the ATC classification system. Zinc plays a critical role in catalytic, structural, and regulatory functions, and its deficiency is associated with taste disorders and impaired cell-mediated immunity (Tuerk and Fazel, 2009; Yagi et al., 2013). The availability of zinc supplementation for chemotherapy-related taste disorders is a topic of debate, indicating that zinc deficiency may contribute to the development of these drug-related taste disorders (Hovan et al., 2010; Hoppe et al., 2021; Ito et al., 2022; Seiki et al., 2024). For most of these antineoplastic and immunomodulating agents, the time-to-onset of taste disorders occurred after several weeks. However, for enzalutamide, palbociclib, and panitumumab, it extended beyond 4 weeks (Table 4). The relatively long onset time for taste disorders associated with enzalutamide, indicated for prostate cancer, aligns with a previous case report and may be related to sex hormones (Kawahara et al., 2019). Palbociclib is used in combination with hormone therapy for breast cancer. These findings suggest that sex hormones may influence taste disorders.

Terbinafine is involved in the development of taste disorders (Beutler et al., 1993; Stricker et al., 1996; Duxbury et al., 2000). The present study confirms this association and reveals that terbinafine-related taste disorders often develop within 4–6 weeks after initiating medication. This was similar to a previous report by Tuccori et al. that used the Italian national database of spontaneous adverse drug reaction reporting (Tuccori et al., 2011). The risk associated with taste disorders may gradually diminish over time. However, the underlying mechanisms remain unclear, as reported previously (Beutler et al., 1993; Duxbury et al., 2000; Tuccori et al., 2011).

Although taste disorders are listed as side effects of varenicline tartrate in package inserts, this association has not been explored in the literature. However, current smoking, particularly with chronicity and/or dependence, is linked to a reduced tongue-tip sensitivity for bitter and salty stimuli (Berube et al., 2021). Therefore, smoking cessation with varenicline restores taste function; however, this altered taste may be perceived as a taste disorder by patients taking varenicline.

The results of this study indicate that drugs associated with taste disorders are predominantly anticancer agents and immunomodulators, although their mechanisms of action are not necessarily identical. It has been confirmed that the tyrosine kinase inhibitors sunitinib malate, pazopanib hydrochloride, and crizotinib can induce taste disorders within a relatively short period, typically within 3 weeks. However, it should be noted that these drugs have different indications and are not all targeted at gastrointestinal tumors. Sunitinib malate and pazopanib hydrochloride are tyrosine kinase inhibitors that specifically target vascular endothelial growth factor receptors (VEGFR). They may affect taste bud cell remodeling by inhibiting the Wnt/β-catenin signaling pathway, where VEGFR1 acts as a positive regulator (Katoh and Katoh, 2006; Naik et al., 2009). This inhibition may lead to the rapid onset of taste disorders associated with these tyrosine kinase inhibitors. Conversely, both romidepsin and vorinostat are histone deacetylase inhibitors. It is believed that the induction of apoptosis associated with changes in various gene expressions contributes to the development of taste disorders. This mechanism is also observed in enfortumab vedotin, which induces apoptosis via binding to microtubules. Fluorouracil and panitumumab are both used for colorectal cancer treatment, but their mechanisms of action are entirely different. Accordingly, while this study identified drugs with a relatively high suspected association with taste disorders using the FAERS and JADER databases, the mechanisms by which these drugs cause such disorders are diverse. Future *in vitro* studies are expected to further elucidate the specific mechanisms through which each drug induces taste disorders.

Age is associated with the prevalence of taste disorders (Hoffman et al., 1998; Bhattacharyya and Kepnes, 2015; Syed et al., 2016; Nin and Tsuzuki, 2024). Additionally, the FAERS database reflected a higher frequency of drug-related taste disorders in females than in males, which may have impacted the signal intensity (Table 2). Therefore, logistic regression analysis was performed to calculate the ROR adjusted for age and sex, revealing that all adjusted RORs were significant for drugs that exhibited a signal in both the FAERS and JADER databases (Table 5). Nirmatrelvir/ritonavir, sunitinib malate, clarithromycin, enzalutamide, palbociclib, pazopanib hydrochloride, terbinafine hydrochloride, crizotinib, fluorouracil, varenicline tartrate, enfortumab vedotin, and panitumumab may be involved in the development of taste disorders, irrespective of age and sex. On the other hand, it should be noted that this model does not consider other potential confounding factors, including concomitant drugs and underlying conditions.

This study utilized signal detection techniques with data from the FAERS and JADER databases, which differ markedly in structure and population. A key advantage of analyzing data from both the FAERS and JADER databases is their external validity, meaning that the results are not confined to a specific region, ethnicity, or set of circumstances. However, the substantial disparity in the size of these databases and their inherent differences require careful interpretation of the results, particularly when a positive signal is detected in one database but not in the other. Such discrepancies may arise from variations in medication usage patterns, genetic backgrounds, reporting requirements, scope of coverage, and reporting accuracy. Therefore, while combining results from the FAERS and JADER databases enhances reliability, it is important to note that significant differences between the two can also increase the risk of false negatives.

SRS-based databases, such as FAERS and JADER, are efficient sources of information for patients treated with drugs. However, analyses using this type of database have certain limitations, including the reporting of duplicates, lack of exposure data, underreporting/overreporting of AEs, notoriety bias, and variable report quality (CIOMS Working Group VIII, 2010; Nomura et al., 2015; Kamimura et al., 2023). This is because the data-collection method in an SRS is generally passive surveillance, mainly by the consumers. Therefore, it is not possible to evaluate the incidence rate or establish a causal relationship from SRS-based databases because of these limitations. However, the data collection method in the JADER database is stimulated by reporting early post-marketing phase vigilance, which is predominantly collected from prescribers or pharmaceutical manufacturers (Table 2) (CIOMS Working Group VIII, 2010). There are differences in the geographical distribution and race of the data between the FAERS and JADER databases. Therefore, the validation of the signal results from the FAERS database with those from the JADER database would compensate for some of the limitations and differences and enhance confidence in the results. On the other hand, the logistic regression model used in this study has certain limitations. Concomitant medications and underlying diseases or conditions can serve as potential confounding factors between specific drugs and adverse events. However, since this study focused on identifying drugs associated with taste disorders, incorporating these factors proved to be challenging. Consequently, concomitant medications and underlying diseases or conditions were not included as covariates in the logistic regression model. Despite these limitations, the findings of this study may be useful for healthcare providers to recognize drug-induced taste disorders, resulting in a reduction in quality of life and noncompliance with medication.

## 5 Conclusion

In summary, this study broadly evaluated the association between various drugs and taste disorders using the FAERS and JADER databases and analyzed their characteristics through the FAERS database. Consequently, signals of drug-related taste disorders were identified for 14 drugs. Among these, 12 were analyzed using a Weibull distribution, which revealed an early failure pattern of taste disorders, with the exception of erythromycin. The same drugs were further analyzed using a logistic regression model, demonstrating a significant relationship with taste disorders, irrespective of age and sex. The results of this study indicate the need for caution regarding taste disorders when administering these drugs. These findings may help minimize the risk of drug-induced taste disorders.

## Data Availability

The original contributions presented in the study are included in the article/Supplementary Material, further inquiries can be directed to the corresponding author.
