# Supplementary material for: Disproportionality analysis of taste disorders using the FDA adverse event reporting system and Japanese adverse drug event report databases
Source: Front Pharmacol. 2025 Nov 27;16:1659746. doi: 10.3389/fphar.2025.1659746 (PMC12695858; doi:10.3389/fphar.2025.1659746)
Supplement: Supplementary file 1 [file Supplementaryfile1.docx]

Supplementary Material

# Supplementary Tables

Table S1. RORs and ICs of drugs associated with taste disorders in both the FAERS database.

| **Drug** | **ATC code** | **Cases** | **Non-cases** | **Total** | **ROR (95%CI)** | **IC (IC_025_)** |
| --- | --- | --- | --- | --- | --- | --- |
| Total |  | 24,485 | 10,152,779 | 10,177,264 |  |  |
| NIRMATRELVIR/RITONAVIR | J05AE30 | 3,968 | 72,107 | 76,075 | 27.04(26.11–27.99) | 4.43(4.38) |
| SODIUM CITRATE | B05CB02 | 1,129 | 29,613 | 30,742 | 16.52(15.55–17.56) | 3.91(3.82) |
| LENALIDOMIDE | L04AX04 | 1,072 | 223,929 | 225,001 | 2.03(1.91–2.16) | 0.98(0.89) |
| CABOZANTINIB S-MALATE | L01EX07 | 858 | 40,308 | 41,166 | 9.11(8.5–9.76) | 3.10(3.00) |
| SUNITINIB MALATE | L01EX01 | 514 | 28,585 | 29,099 | 7.59(6.95–8.29) | 2.86(2.73) |
| VISMODEGIB | L01XJ01 | 481 | 5,320 | 5,801 | 38.22(34.79–42.0) | 5.01(4.87) |
| CLARITHROMYCIN | J01FA09 | 419 | 14,487 | 14,906 | 12.18(11.05–13.44) | 3.51(3.37) |
| ENZALUTAMIDE | L02BB04 | 358 | 51,749 | 52,107 | 2.90(2.61–3.22) | 1.51(1.35) |
| ADALIMUMAB | L04AB04 | 293 | 365,483 | 365,776 | 0.32(0.29–0.36) | -1.58(-1.75) |
| LIFITEGRAST | S01XA25 | 261 | 4,930 | 5,191 | 22.18(19.57–25.13) | 4.28(4.10) |
| PALBOCICLIB | L01EF01 | 259 | 67,521 | 67,780 | 1.60(1.41–1.81) | 0.66(0.48) |
| NINTEDANIB | L01EX09 | 256 | 46,212 | 46,468 | 2.31(2.04–2.61) | 1.19(1.01) |
| TERBINAFINE | D01AE15 | 232 | 3,386 | 3,618 | 28.67(25.09–32.77) | 4.59(4.39) |
| NIRAPARIB | L01XK02 | 207 | 35,808 | 36,015 | 2.41(2.1–2.76) | 1.25(1.05) |
| SECUKINUMAB | L04AC10 | 202 | 118,231 | 118,433 | 0.71(0.61–0.81) | -0.49(-0.7) |
| FINGOLIMOD HYDROCHLORIDE | L04AE01 | 200 | 99,765 | 99,965 | 0.83(0.72–0.95) | -0.26(-0.47) |
| PIRFENIDONE | L04AX05 | 195 | 27,448 | 27,643 | 2.96(2.57–3.41) | 1.54(1.33) |
| TERIPARATIDE | H05AA02 | 184 | 69,756 | 69,940 | 1.09(0.95–1.27) | 0.13(-0.09) |
| OMEPRAZOLE | A02BC01 | 174 | 14,763 | 14,937 | 4.92(4.23–5.71) | 2.24(2.02) |
| EVEROLIMUS | L01EG02 | 172 | 38,229 | 38,401 | 1.87(1.61–2.18) | 0.89(0.67) |
| NIVOLUMAB | L01FF01 | 165 | 87,442 | 87,607 | 0.78(0.67–0.91) | -0.35(-0.58) |
| RUCAPARIB CAMSYLATE | L01XK03 | 162 | 7,527 | 7,689 | 8.98(7.68–10.49) | 3.06(2.83) |
| AVAPRITINIB | L01EX18 | 162 | 16,101 | 16,263 | 4.19(3.59–4.90) | 2.02(1.79) |
| SEMAGLUTIDE | A10BJ06 | 156 | 30,206 | 30,362 | 2.15(1.84–2.52) | 1.08(0.85) |
| PAZOPANIB HYDROCHLORIDE | L01EX03 | 155 | 18,404 | 18,559 | 3.51(2.99–4.11) | 1.77(1.54) |
| DUPILUMAB | D11AH05 | 149 | 159,185 | 159,334 | 0.38(0.33–0.45) | -1.36(-1.59) |
| METRONIDAZOLE | A01AB17 | 148 | 9,670 | 9,818 | 6.38(5.42–7.51) | 2.60(2.36) |
| CAPECITABINE | L01BC06 | 145 | 36,518 | 36,663 | 1.65(1.40–1.94) | 0.71(0.47) |
| APREMILAST | L04AA32 | 133 | 85,195 | 85,328 | 0.65(0.54–0.77) | -0.62(-0.87) |
| AZELASTINE HYDROCHLORIDE | R01AC03 | 117 | 1,659 | 1,776 | 29.38(24.35–35.45) | 4.48(4.21) |
| OCRELIZUMAB | L04AG08 | 113 | 82,112 | 82,225 | 0.57(0.47–0.68) | -0.8(-1.07) |
| FLUTICASONE PROPIONATE | D07AC17 | 110 | 7,593 | 7,703 | 6.03(4.99–7.28) | 2.51(2.23) |
| POMALIDOMIDE | L04AX06 | 105 | 54,615 | 54,720 | 0.8(0.66–0.96) | -0.32(-0.6) |
| TERBINAFINE HYDROCHLORIDE | D01AE15 | 104 | 1,645 | 1,749 | 26.32(21.58–32.1) | 4.33(4.04) |
| PACLITAXEL | L01CD01 | 98 | 37,952 | 38,050 | 1.07(0.88–1.31) | 0.1(-0.19) |
| ETANERCEPT | L04AB01 | 97 | 145,140 | 145,237 | 0.27(0.22–0.33) | -1.84(-2.13) |
| TREPROSTINIL | B01AC21 | 95 | 91,884 | 91,979 | 0.43(0.35–0.52) | -1.21(-1.51) |
| LENVATINIB | L01EX08 | 91 | 30,539 | 30,630 | 1.24(1.01–1.52) | 0.30(-0.00) |
| PEMBROLIZUMAB | L01FF02 | 91 | 47,578 | 47,669 | 0.79(0.64–0.97) | -0.33(-0.63) |
| CIPROFLOXACIN | J01MA02 | 91 | 40,942 | 41,033 | 0.92(0.75–1.13) | -0.12(-0.42) |
| DULAGLUTIDE | A10BJ05 | 89 | 27,407 | 27,496 | 1.35(1.09–1.66) | 0.42(0.12) |
| SERTRALINE HYDROCHLORIDE | N06AB06 | 86 | 35,665 | 35,751 | 1.0(0.81–1.24) | -0.0(-0.31) |
| SODIUM OXYBATE | N01AX11 | 85 | 57,853 | 57,938 | 0.61(0.49–0.75) | -0.71(-1.02) |
| RIBOCICLIB | L01EF02 | 82 | 28,759 | 28,841 | 1.18(0.95–1.47) | 0.24(-0.08) |
| APOMORPHINE HYDROCHLORIDE | N04BC07 | 80 | 11,340 | 11,420 | 2.93(2.35–3.65) | 1.51(1.19) |
| NICOTINE | N07BA01 | 77 | 13,722 | 13,799 | 2.33(1.86–2.92) | 1.19(0.86) |
| CRIZOTINIB | L01ED01 | 76 | 8,697 | 8,773 | 3.63(2.90–4.55) | 1.80(1.47) |
| LEVOFLOXACIN | J01MA12 | 75 | 31,217 | 31,292 | 1.00(0.79–1.25) | -0.01(-0.34) |
| TOFACITINIB CITRATE | L04AF01 | 71 | 79,213 | 79,284 | 0.37(0.29–0.47) | -1.41(-1.75) |
| DIMETHYL FUMARATE | L04AX07 | 70 | 72,114 | 72,184 | 0.40(0.32–0.51) | -1.3(-1.64) |
| OMALIZUMAB | R03DX05 | 69 | 88,764 | 88,833 | 0.32(0.25–0.41) | -1.62(-1.96) |
| TOPIRAMATE | N03AX11 | 68 | 8,570 | 8,638 | 3.30(2.6–4.19) | 1.66(1.31) |
| OXALIPLATIN | L01XA03 | 67 | 35,513 | 35,580 | 0.78(0.61–0.99) | -0.35(-0.70) |
| EVOLOCUMAB | C10AX13 | 66 | 58,835 | 58,901 | 0.46(0.36–0.59) | -1.09(-1.44) |
| OFATUMUMAB | L01FA02 | 65 | 39,829 | 39,894 | 0.68(0.53–0.86) | -0.56(-0.91) |
| PEGINTERFERON ALFA-2A | L03AB11 | 64 | 11,206 | 11,270 | 2.37(1.85–3.03) | 1.21(0.85) |
| CARBOPLATIN | L01XA02 | 64 | 39,327 | 39,391 | 0.67(0.53–0.86) | -0.56(-0.92) |
| ALBUTEROL SULFATE | R03AC02 | 64 | 8,391 | 8,455 | 3.17(2.48–4.05) | 1.61(1.25) |
| GLATIRAMER ACETATE | L03AX13 | 63 | 35,512 | 35,575 | 0.73(0.57–0.94) | -0.44(-0.8) |
| ERLOTINIB HYDROCHLORIDE | L01EB02 | 63 | 10,571 | 10,634 | 2.48(1.93–3.17) | 1.27(0.91) |
| SACUBITRIL\VALSARTAN | C09DX04 | 61 | 57,074 | 57,135 | 0.44(0.34–0.57) | -1.16(-1.53) |
| TERIFLUNOMIDE | L04AK02 | 61 | 44,349 | 44,410 | 0.57(0.44–0.73) | -0.8(-1.17) |
| ENFORTUMAB VEDOTIN-EJFV | L01FX13 | 61 | 2,401 | 2,462 | 10.56(8.19–13.62) | 3.16(2.79) |
| POLYETHYLENE GLYCOL 3350 | A06AD15 | 61 | 14,948 | 15,009 | 1.69(1.32–2.18) | 0.74(0.37) |
| LIRAGLUTIDE | A10BJ02 | 59 | 13,294 | 13,353 | 1.84(1.43–2.38) | 0.86(0.48) |
| DOCETAXEL | L01CD02 | 58 | 64,947 | 65,005 | 0.37(0.28–0.48) | -1.42(-1.79) |
| FLUOROURACIL | L01BC02 | 57 | 12,833 | 12,890 | 1.84(1.42–2.39) | 0.86(0.48) |
| VEDOLIZUMAB | L04AG05 | 56 | 116,590 | 116,646 | 0.2(0.15–0.26) | -2.3(-2.69) |
| IBRUTINIB | L01EL01 | 56 | 59,366 | 59,422 | 0.39(0.3–0.51) | -1.34(-1.72) |
| AMLODIPINE BESYLATE | C08CA01 | 55 | 18,547 | 18,602 | 1.23(0.94–1.6) | 0.29(-0.10) |
| ABEMACICLIB | L01EF03 | 55 | 9,523 | 9,578 | 2.4(1.84–3.13) | 1.22(0.83) |
| IBUPROFEN | C01EB16 | 55 | 28,594 | 28,649 | 0.8(0.61–1.04) | -0.32(-0.71) |
| CERTOLIZUMAB PEGOL | L04AB05 | 55 | 63,334 | 63,389 | 0.36(0.28–0.47) | -1.45(-1.84) |
| ABALOPARATIDE | H05AA04 | 54 | 19,769 | 19,823 | 1.13(0.87–1.48) | 0.18(-0.21) |
| AFATINIB | L01EB03 | 54 | 11,211 | 11,265 | 2.0(1.53–2.61) | 0.97(0.58) |
| DENOSUMAB | M05BX04 | 53 | 45,547 | 45,600 | 0.48(0.37–0.63) | -1.04(-1.43) |
| LEUPROLIDE ACETATE | L02AE02 | 53 | 63,296 | 63,349 | 0.35(0.26–0.45) | -1.51(-1.9) |
| ZOLEDRONIC ACID | M05BA08 | 53 | 29,316 | 29,369 | 0.75(0.57–0.98) | -0.41(-0.8) |
| MACITENTAN | C02KX04 | 53 | 82,950 | 83,003 | 0.26(0.2–0.34) | -1.89(-2.29) |
| VARENICLINE TARTRATE | N07BA03 | 52 | 9,353 | 9,405 | 2.31(1.76–3.03) | 1.17(0.77) |
| CARBIDOPA\LEVODOPA | N04BA02 | 52 | 70,294 | 70,346 | 0.31(0.23–0.4) | -1.68(-2.08) |
| TRASTUZUMAB | L01FD01 | 50 | 19,992 | 20,042 | 1.04(0.79–1.37) | 0.05(-0.35) |
| AZELASTINE HYDROCHLORIDE\FLUTICASONE PROPIONATE | #N/A | 47 | 963 | 1,010 | 20.27(15.12–27.18) | 3.81(3.38) |
| CHLORHEXIDINE GLUCONATE | A01AB03 | 44 | 1,319 | 1,363 | 13.86(10.26–18.71) | 3.39(2.96) |
| OCTREOTIDE ACETATE | H01CB02 | 44 | 57,170 | 57,214 | 0.32(0.24–0.43) | -1.62(-2.05) |
| RIVAROXABAN | B01AF01 | 44 | 88,868 | 88,912 | 0.2(0.15–0.27) | -2.26(-2.69) |
| DICHLORPHENAMIDE | S01EC02 | 44 | 2,390 | 2,434 | 7.65(5.67–10.3) | 2.71(2.28) |
| BEVACIZUMAB | L01FG01 | 44 | 38,130 | 38,174 | 0.48(0.36–0.64) | -1.04(-1.48) |
| MARIBAVIR | J05AX10 | 43 | 652 | 695 | 27.39(20.11–37.31) | 4.04(3.59) |
| IMATINIB MESYLATE | L01EA01 | 43 | 17,642 | 17,685 | 1.01(0.75–1.36) | 0.01(-0.42) |
| TIRZEPATIDE | A10BX16 | 42 | 22,291 | 22,333 | 0.78(0.58–1.06) | -0.35(-0.79) |
| NALTREXONE | N07BB04 | 42 | 21,602 | 21,644 | 0.81(0.6–1.09) | -0.3(-0.74) |
| FLUTICASONE FUROATE\VILANTEROL TRIFENATATE | R03AK10 | 42 | 9,513 | 9,555 | 1.83(1.35–2.48) | 0.84(0.4) |
| CETYLPYRIDINIUM CHLORIDE | D08AJ03 | 42 | 224 | 266 | 77.88(56.0–108.31) | 4.71(4.24) |
| ESKETAMINE | N01AX14 | 40 | 7,807 | 7,847 | 2.13(1.56–2.9) | 1.04(0.59) |
| UMECLIDINIUM BROMIDE\VILANTEROL TRIFENATATE | R03AL03 | 40 | 5,638 | 5,678 | 2.95(2.16–4.02) | 1.48(1.03) |
| ALPELISIB | L01EM03 | 40 | 6,437 | 6,477 | 2.58(1.89–3.52) | 1.31(0.85) |
| LETROZOLE | L02BG04 | 40 | 18,081 | 18,121 | 0.92(0.67–1.25) | -0.12(-0.57) |
| ENFORTUMAB VEDOTIN | L01FX13 | 39 | 1,983 | 2,022 | 8.17(5.95–11.21) | 2.77(2.31) |
| APIXABAN | B01AF02 | 39 | 46,849 | 46,888 | 0.34(0.25–0.47) | -1.51(-1.97) |
| OSELTAMIVIR PHOSPHATE | J05AH02 | 39 | 7,157 | 7,196 | 2.26(1.65–3.1) | 1.13(0.67) |
| DULOXETINE HYDROCHLORIDE | N06AX21 | 39 | 21,102 | 21,141 | 0.77(0.56–1.05) | -0.37(-0.83) |
| MAGNESIUM SULFATE\POTASSIUM SULFATE\SODIUM SULFATE | #N/A | 38 | 6,792 | 6,830 | 2.32(1.69–3.19) | 1.16(0.7) |
| SORAFENIB | L01EX02 | 38 | 18,568 | 18,606 | 0.85(0.62–1.17) | -0.23(-0.69) |
| FLUTICASONE PROPIONATE\SALMETEROL XINAFOATE | R03AK06 | 38 | 9,466 | 9,504 | 1.67(1.21–2.29) | 0.71(0.25) |
| CYCLOSPORINE | S01XA18 | 38 | 14,814 | 14,852 | 1.06(0.77–1.46) | 0.09(-0.38) |
| ENTRECTINIB | L01EX14 | 38 | 1,252 | 1,290 | 12.6(9.12–17.41) | 3.25(2.78) |
| BUDESONIDE | A07EA06 | 37 | 8,430 | 8,467 | 1.82(1.32–2.52) | 0.83(0.36) |
| APALUTAMIDE | L02BB05 | 37 | 4,718 | 4,755 | 3.26(2.36–4.5) | 1.61(1.14) |
| REGORAFENIB | L01EX05 | 37 | 18,836 | 18,873 | 0.81(0.59–1.12) | -0.29(-0.76) |
| ERENUMAB-AOOE | N02CD01 | 36 | 28,439 | 28,475 | 0.52(0.38–0.73) | -0.91(-1.38) |
| AXITINIB | L01EK01 | 36 | 10,589 | 10,625 | 1.41(1.02–1.96) | 0.48(0.0) |
| VEMURAFENIB | L01EC01 | 36 | 6,450 | 6,486 | 2.32(1.67–3.21) | 1.16(0.68) |
| LEVOTHYROXINE SODIUM | H03AA01 | 36 | 41,357 | 41,393 | 0.36(0.26–0.5) | -1.44(-1.92) |
| ONABOTULINUMTOXINA | #N/A | 35 | 25,327 | 25,362 | 0.57(0.41–0.8) | -0.78(-1.27) |
| OXYMETAZOLINE HYDROCHLORIDE | R01AA05 | 35 | 2,268 | 2,303 | 6.41(4.59–8.95) | 2.46(1.97) |
| AZITHROMYCIN ANHYDROUS | #N/A | 35 | 5,847 | 5,882 | 2.48(1.78–3.46) | 1.25(0.77) |
| FLUTICASONE FUROATE\UMECLIDINIUM BROMIDE\VILANTEROL TRIFENATATE | R03AL08 | 34 | 5,007 | 5,041 | 2.82(2.01–3.95) | 1.41(0.92) |
| DICLOFENAC SODIUM | M01AB05? | 33 | 16,056 | 16,089 | 0.85(0.61–1.2) | -0.22(-0.72) |
| PANITUMUMAB | L01FE02 | 33 | 7,525 | 7,558 | 1.82(1.29–2.56) | 0.83(0.33) |
| TIPIRACIL HYDROCHLORIDE\TRIFLURIDINE | L01BC59 | 33 | 8,380 | 8,413 | 1.63(1.16–2.3) | 0.68(0.18) |
| PHENTERMINE HYDROCHLORIDE\TOPIRAMATE | A08AA51 | 32 | 1,877 | 1,909 | 7.08(4.99–10.04) | 2.56(2.05) |
| NATALIZUMAB | L04AG03 | 32 | 59,020 | 59,052 | 0.22(0.16–0.32) | -2.12(-2.62) |
| FLUTICASONE FUROATE | R01AD12 | 32 | 2,124 | 2,156 | 6.25(4.41–8.87) | 2.41(1.91) |
| DASABUVIR\OMBITASVIR\PARITAPREVIR\RITONAVIR | J05AP52 | 32 | 14,454 | 14,486 | 0.92(0.65–1.3) | -0.12(-0.62) |
| METFORMIN HYDROCHLORIDE | A10BA02 | 31 | 17,173 | 17,204 | 0.75(0.53–1.06) | -0.41(-0.92) |
| LINEZOLID | J01XX08 | 31 | 8,905 | 8,936 | 1.44(1.01–2.06) | 0.51(-0.0) |
| LEVONORGESTREL | G03AC03 | 31 | 127,796 | 127,827 | 0.1(0.07–0.14) | -3.27(-3.78) |
| CLOPIDOGREL BISULFATE | B01AC04 | 30 | 18,135 | 18,165 | 0.69(0.48–0.98) | -0.53(-1.05) |
| DABRAFENIB MESYLATE | L01EC02 | 30 | 12,566 | 12,596 | 0.99(0.69–1.42) | -0.01(-0.53) |
| TIOTROPIUM BROMIDE MONOHYDRATE | R03BB04 | 30 | 10,439 | 10,469 | 1.19(0.83–1.71) | 0.24(-0.28) |
| ABIRATERONE ACETATE | L02BX03 | 30 | 13,878 | 13,908 | 0.9(0.63–1.28) | -0.15(-0.67) |
| ATORVASTATIN | C10AA05 | 30 | 18,281 | 18,311 | 0.68(0.48–0.97) | -0.54(-1.06) |
| RITUXIMAB | L01FA01 | 30 | 50,661 | 50,691 | 0.24(0.17–0.35) | -1.99(-2.51) |
| CORTICOTROPIN | H01AA01 | 30 | 11,404 | 11,434 | 1.09(0.76–1.56) | 0.12(-0.4) |
| HUMAN IMMUNOGLOBULIN G | #N/A | 29 | 93,469 | 93,498 | 0.13(0.09–0.18) | -2.91(-3.44) |
| OSIMERTINIB | L01EB04 | 29 | 6,022 | 6,051 | 2.0(1.39–2.88) | 0.95(0.42) |
| ABATACEPT | L04AA24 | 29 | 54,740 | 54,769 | 0.22(0.15–0.31) | -2.15(-2.67) |
| AMBRISENTAN | C02KX02 | 28 | 61,290 | 61,318 | 0.19(0.13–0.27) | -2.36(-2.89) |
| AMIKACIN | D06AX12 | 28 | 3,330 | 3,358 | 3.49(2.41–5.06) | 1.68(1.14) |
| ATEZOLIZUMAB | L01FF05 | 28 | 26,588 | 26,616 | 0.44(0.3–0.63) | -1.17(-1.7) |
| PRALSETINIB | L01EX23 | 28 | 1,832 | 1,860 | 6.34(4.37–9.22) | 2.4(1.86) |
| AMOXICILLIN | J01CA04 | 28 | 17,026 | 17,054 | 0.68(0.47–0.99) | -0.54(-1.07) |
| IXAZOMIB | L01XG03 | 28 | 13,321 | 13,349 | 0.87(0.6–1.26) | -0.19(-0.73) |
| CITALOPRAM HYDROBROMIDE | N06AB04 | 27 | 10,474 | 10,501 | 1.07(0.73–1.56) | 0.09(-0.45) |
| LEDIPASVIR\SOFOSBUVIR | J05AP51 | 27 | 18,357 | 18,384 | 0.61(0.42–0.89) | -0.69(-1.24) |
| ACETAMINOPHEN | N02BE01 | 27 | 28,418 | 28,445 | 0.39(0.27–0.57) | -1.31(-1.86) |
| ZINC ACETATE ANHYDROUS\ZINC GLUCONATE | #N/A | 27 | 28 | 55 | 400.28(235.89–679.25) | 4.63(3.96) |
| TEPROTUMUMAB-TRBW | L04AG13 | 27 | 2,546 | 2,573 | 4.4(3.01–6.43) | 1.96(1.41) |
| CLINDAMYCIN | D10AF01 | 27 | 3,517 | 3,544 | 3.19(2.18–4.65) | 1.56(1.01) |
| VENETOCLAX | L01XX52 | 26 | 33,923 | 33,949 | 0.32(0.22–0.47) | -1.61(-2.17) |
| OLAPARIB | L01XK01 | 26 | 4,373 | 4,399 | 2.47(1.68–3.63) | 1.22(0.66) |
| IRINOTECAN | L01CE02 | 26 | 6,026 | 6,052 | 1.79(1.22–2.63) | 0.79(0.24) |
| INSULIN GLARGINE | A10AE04 | 26 | 16,721 | 16,747 | 0.64(0.44–0.95) | -0.61(-1.17) |
| ALEMTUZUMAB | L04AG06 | 26 | 25,493 | 25,519 | 0.42(0.29–0.62) | -1.21(-1.76) |
| FENTANYL | N01AH01 | 26 | 10,116 | 10,142 | 1.07(0.73–1.57) | 0.09(-0.47) |
| NILOTINIB | L01EA03 | 26 | 25,293 | 25,319 | 0.43(0.29–0.63) | -1.2(-1.75) |
| RISANKIZUMAB-RZAA | L04AC18 | 25 | 31,434 | 31,459 | 0.33(0.22–0.49) | -1.56(-2.13) |
| SODIUM FLUORIDE | A01AA01 | 25 | 799 | 824 | 12.99(8.72–19.34) | 3.12(2.55) |
| CABOZANTINIB | L01EX07 | 25 | 4,008 | 4,033 | 2.59(1.75–3.84) | 1.28(0.71) |
| IXAZOMIB CITRATE | L01XG03 | 25 | 6,564 | 6,589 | 1.58(1.07–2.34) | 0.63(0.06) |
| INTERFERON BETA-1A | L03AB07 | 25 | 50,507 | 50,532 | 0.2(0.14–0.3) | -2.24(-2.8) |
| MOMETASONE FUROATE | R03BA07 | 25 | 4,623 | 4,648 | 2.24(1.51–3.32) | 1.09(0.53) |
| DEXAMETHASONE | A01AC02 | 24 | 17,305 | 17,329 | 0.57(0.38–0.86) | -0.77(-1.35) |
| BAMLANIVIMAB | #N/A | 24 | 11,020 | 11,044 | 0.9(0.6–1.35) | -0.14(-0.72) |
| VENLAFAXINE HYDROCHLORIDE | N06AX16 | 24 | 19,818 | 19,842 | 0.5(0.34–0.75) | -0.96(-1.54) |
| DASATINIB | L01EA02 | 24 | 13,085 | 13,109 | 0.76(0.51–1.13) | -0.38(-0.96) |
| RIBAVIRIN | J05AP01 | 24 | 11,020 | 11,044 | 0.9(0.6–1.35) | -0.14(-0.72) |
| CETUXIMAB | L01FE01 | 24 | 11,947 | 11,971 | 0.83(0.56–1.24) | -0.25(-0.83) |
| AMPHETAMINE ASPARTATE\AMPHETAMINE SULFATE\DEXTROAMPHETAMINE SACCHARATE\DEXTROAMPHETAMINE SULFATE | #N/A | 24 | 11,237 | 11,261 | 0.89(0.59–1.32) | -0.17(-0.75) |
| ALIROCUMAB | C10AX14 | 24 | 15,758 | 15,782 | 0.63(0.42–0.94) | -0.64(-1.22) |
| ESZOPICLONE | N05CF04 | 23 | 619 | 642 | 15.42(10.17–23.39) | 3.24(2.64) |
| ALECTINIB HYDROCHLORIDE | L01ED03 | 23 | 3,779 | 3,802 | 2.53(1.68–3.81) | 1.24(0.65) |
| TOCILIZUMAB | L04AC07 | 23 | 51,150 | 51,173 | 0.19(0.12–0.28) | -2.37(-2.96) |
| SOFOSBUVIR\VELPATASVIR | J05AP55 | 23 | 13,526 | 13,549 | 0.7(0.47–1.06) | -0.49(-1.08) |
| UPADACITINIB | L04AF03 | 22 | 34,761 | 34,783 | 0.26(0.17–0.4) | -1.88(-2.48) |
| LORATADINE | R06AX13 | 22 | 9,481 | 9,503 | 0.96(0.63–1.46) | -0.05(-0.66) |
| FERRIC CARBOXYMALTOSE | #N/A | 22 | 10,953 | 10,975 | 0.83(0.55–1.27) | -0.25(-0.86) |
| DEXTROMETHORPHAN HYDROBROMIDE\GUAIFENESIN | #N/A | 22 | 5,046 | 5,068 | 1.81(1.19–2.75) | 0.8(0.2) |
| GADOTERATE MEGLUMINE | V08CA02 | 22 | 7,564 | 7,586 | 1.21(0.79–1.83) | 0.26(-0.35) |
| CLINDAMYCIN HYDROCHLORIDE | J01FF01 | 22 | 2,251 | 2,273 | 4.06(2.66–6.17) | 1.83(1.22) |
| CETIRIZINE HYDROCHLORIDE | S01GX12 | 22 | 10,967 | 10,989 | 0.83(0.55–1.26) | -0.25(-0.86) |
| HOMEOPATHICS\ZINC ACETATE\ZINC GLUCONATE | #N/A | 22 | 55 | 77 | 166.01(101.24–272.23) | 4.28(3.59) |
| VORTIOXETINE HYDROBROMIDE | N06AX26 | 22 | 5,912 | 5,934 | 1.54(1.02–2.35) | 0.59(-0.01) |
| SELEXIPAG | B01AC27 | 21 | 22,337 | 22,358 | 0.39(0.25–0.6) | -1.32(-1.93) |
| ESOMEPRAZOLE MAGNESIUM | A02BC05 | 21 | 5,822 | 5,843 | 1.5(0.97–2.3) | 0.55(-0.07) |
| GABAPENTIN | N02BF01 | 21 | 18,591 | 18,612 | 0.47(0.3–0.72) | -1.06(-1.67) |
| LISINOPRIL | C09AA03 | 21 | 12,040 | 12,061 | 0.72(0.47–1.11) | -0.45(-1.06) |
| NAPROXEN SODIUM | M01AE02 | 21 | 6,942 | 6,963 | 1.25(0.82–1.93) | 0.31(-0.31) |
| PREGABALIN | N02BF02 | 21 | 33,256 | 33,277 | 0.26(0.17–0.4) | -1.88(-2.5) |
| EMPAGLIFLOZIN | A10BK03 | 21 | 18,128 | 18,149 | 0.48(0.31–0.74) | -1.02(-1.64) |
| GOLIMUMAB | L04AB06 | 21 | 31,011 | 31,032 | 0.28(0.18–0.43) | -1.78(-2.4) |
| MIRTAZAPINE | N06AX11 | 21 | 12,519 | 12,540 | 0.7(0.45–1.07) | -0.5(-1.12) |
| CIPROFLOXACIN HYDROCHLORIDE | J01MA02 | 21 | 9,271 | 9,292 | 0.94(0.61–1.44) | -0.09(-0.7) |
| RIOCIGUAT | C02KX05 | 20 | 21,649 | 21,669 | 0.38(0.25–0.59) | -1.34(-1.97) |
| LANSOPRAZOLE | A02BC03 | 20 | 8,512 | 8,532 | 0.97(0.63–1.51) | -0.04(-0.67) |
| PREDNISOLONE | A01AC04 | 20 | 8,684 | 8,704 | 0.95(0.62–1.48) | -0.06(-0.69) |
| ZINC ACETATE\ZINC GLUCONATE | #N/A | 20 | 34 | 54 | 244.11(140.49–424.16) | 4.21(3.47) |
| DALFAMPRIDINE | N07XX07 | 20 | 41,688 | 41,708 | 0.2(0.13–0.31) | -2.27(-2.9) |
| BELIMUMAB | L04AG04 | 19 | 14,969 | 14,988 | 0.53(0.34–0.82) | -0.89(-1.54) |
| ERLOTINIB | L01EB02 | 19 | 6,204 | 6,223 | 1.27(0.81–1.99) | 0.32(-0.32) |
| IRINOTECAN HYDROCHLORIDE | L01CE02 | 19 | 5,767 | 5,786 | 1.37(0.87–2.14) | 0.42(-0.22) |
| ALBUTEROL SULFATE\IPRATROPIUM BROMIDE | R03AL02 | 19 | 2,123 | 2,142 | 3.71(2.36–5.83) | 1.7(1.05) |
| LOSARTAN POTASSIUM | C09CA01 | 19 | 3,436 | 3,455 | 2.29(1.46–3.6) | 1.1(0.46) |
| BUPRENORPHINE HYDROCHLORIDE\NALOXONE HYDROCHLORIDE | #N/A | 19 | 7,252 | 7,271 | 1.09(0.69–1.7) | 0.11(-0.53) |
| IXEKIZUMAB | L04AC13 | 19 | 11,227 | 11,246 | 0.7(0.45–1.1) | -0.49(-1.13) |
| LEVODOPA | N04BA01 | 19 | 6,235 | 6,254 | 1.26(0.81–1.98) | 0.32(-0.33) |
| GADOBUTROL | V08CA09 | 19 | 7,449 | 7,468 | 1.06(0.67–1.66) | 0.08(-0.57) |
| MEPOLIZUMAB | R03DX09 | 19 | 18,080 | 18,099 | 0.44(0.28–0.68) | -1.16(-1.8) |
| BUPROPION HYDROCHLORIDE | N06AX12 | 19 | 5,981 | 6,000 | 1.32(0.84–2.07) | 0.37(-0.27) |
| UMECLIDINIUM BROMIDE | R03BB07 | 18 | 1,764 | 1,782 | 4.23(2.66–6.74) | 1.84(1.18) |
| SONIDEGIB | L01XJ02 | 18 | 910 | 928 | 8.21(5.15–13.09) | 2.55(1.89) |
| FEXOFENADINE HYDROCHLORIDE | R06AX26 | 18 | 3,284 | 3,302 | 2.27(1.43–3.61) | 1.09(0.42) |
| INFLIXIMAB | L04AB02 | 18 | 57,616 | 57,634 | 0.13(0.08–0.2) | -2.88(-3.54) |
| MOBOCERTINIB | L01EB10 | 18 | 2,374 | 2,392 | 3.15(1.98–5.0) | 1.49(0.83) |
| ARIPIPRAZOLE | N05AX12 | 18 | 30,189 | 30,207 | 0.25(0.16–0.39) | -1.96(-2.62) |
| MOLNUPIRAVIR | J05AB18 | 18 | 4,755 | 4,773 | 1.57(0.99–2.49) | 0.61(-0.06) |
| TRIAMCINOLONE ACETONIDE | H02AB08 | 18 | 4,331 | 4,349 | 1.72(1.08–2.74) | 0.73(0.06) |
| DOXYCYCLINE | A01AB22 | 17 | 8,444 | 8,461 | 0.83(0.52–1.34) | -0.25(-0.93) |
| RAMIPRIL | C09AA05 | 17 | 9,972 | 9,989 | 0.71(0.44–1.14) | -0.48(-1.16) |
| DEFERASIROX | V03AC03 | 17 | 10,405 | 10,422 | 0.68(0.42–1.09) | -0.53(-1.22) |
| IPILIMUMAB | L01FX04 | 17 | 17,518 | 17,535 | 0.4(0.25–0.65) | -1.26(-1.94) |
| FINASTERIDE | D11AX10 | 17 | 10,777 | 10,794 | 0.65(0.41–1.05) | -0.58(-1.26) |
| RUXOLITINIB | D11AH09 | 17 | 18,738 | 18,755 | 0.38(0.23–0.6) | -1.36(-2.04) |
| CYCLOPHOSPHAMIDE | L01AA01 | 17 | 14,077 | 14,094 | 0.5(0.31–0.81) | -0.96(-1.64) |
| EPIRUBICIN | L01DB03 | 17 | 3,568 | 3,585 | 1.98(1.23–3.18) | 0.9(0.22) |
| BORTEZOMIB | L01XG01 | 16 | 12,135 | 12,151 | 0.55(0.33–0.89) | -0.83(-1.53) |
| GUAIFENESIN | R05CA03 | 16 | 4,055 | 4,071 | 1.64(1.0–2.67) | 0.65(-0.05) |
| MINOXIDIL | C02DC01 | 16 | 11,375 | 11,391 | 0.58(0.36–0.95) | -0.74(-1.44) |
| PERFLUTREN | V08DA01 | 16 | 7,680 | 7,696 | 0.86(0.53–1.41) | -0.2(-0.9) |
| PERTUZUMAB | L01FD02 | 16 | 7,135 | 7,151 | 0.93(0.57–1.52) | -0.1(-0.8) |
| BUPRENORPHINE | N02AE01 | 16 | 10,467 | 10,483 | 0.63(0.39–1.03) | -0.63(-1.33) |
| RANITIDINE HYDROCHLORIDE | A02BA02 | 16 | 217,844 | 217,860 | 0.03(0.02–0.05) | -4.95(-5.65) |
| INSULIN LISPRO | A10AB04 | 15 | 10,819 | 10,834 | 0.57(0.35–0.95) | -0.76(-1.48) |
| FULVESTRANT | L02BA03 | 15 | 4,878 | 4,893 | 1.28(0.77–2.12) | 0.32(-0.4) |
| BUPROPION HYDROCHLORIDE\NALTREXONE HYDROCHLORIDE | #N/A | 15 | 4,818 | 4,833 | 1.29(0.78–2.14) | 0.34(-0.38) |
| IPRATROPIUM BROMIDE | R01AX03 | 15 | 1,330 | 1,345 | 4.68(2.81–7.78) | 1.92(1.19) |
| HOMEOPATHICS | #N/A | 15 | 676 | 691 | 9.21(5.52–15.36) | 2.59(1.86) |
| TADALAFIL | G04BE08 | 15 | 11,425 | 11,440 | 0.54(0.33–0.9) | -0.83(-1.56) |
| BUDESONIDE\FORMOTEROL FUMARATE DIHYDRATE | R03AK07 | 15 | 1,487 | 1,502 | 4.18(2.52–6.96) | 1.79(1.07) |
| PEGFILGRASTIM | L03AA13 | 14 | 8,829 | 8,843 | 0.66(0.39–1.11) | -0.57(-1.32) |
| IBANDRONATE SODIUM | M05BA06 | 14 | 5,013 | 5,027 | 1.16(0.69–1.96) | 0.2(-0.55) |
| ESCITALOPRAM OXALATE | N06AB10 | 14 | 11,455 | 11,469 | 0.51(0.3–0.86) | -0.93(-1.68) |
| MYCOPHENOLATE MOFETIL | L04AA06 | 14 | 7,755 | 7,769 | 0.75(0.44–1.26) | -0.39(-1.14) |
| INSULIN DEGLUDEC | A10AE06 | 14 | 4,693 | 4,707 | 1.24(0.73–2.09) | 0.28(-0.46) |
| LAMOTRIGINE | N03AX09 | 14 | 19,914 | 19,928 | 0.29(0.17–0.49) | -1.71(-2.45) |
| ALENDRONATE SODIUM | M05BA04 | 14 | 5,500 | 5,514 | 1.06(0.62–1.78) | 0.07(-0.67) |
| BOCEPREVIR | J05AP03 | 14 | 483 | 497 | 12.03(7.07–20.46) | 2.77(2.01) |
| ANASTROZOLE | L02BG03 | 14 | 5,296 | 5,310 | 1.1(0.65–1.85) | 0.12(-0.62) |
| PANTOPRAZOLE | A02BC02 | 14 | 6,813 | 6,827 | 0.85(0.5–1.44) | -0.22(-0.96) |
| AMOXICILLIN\CLAVULANATE POTASSIUM | J01RA01 | 13 | 5,878 | 5,891 | 0.92(0.53–1.58) | -0.12(-0.89) |
| FREMANEZUMAB-VFRM | N02CD03 | 13 | 5,059 | 5,072 | 1.07(0.62–1.84) | 0.08(-0.69) |
| BOSENTAN | C02KX01 | 13 | 17,935 | 17,948 | 0.3(0.17–0.52) | -1.66(-2.43) |
| AMIODARONE HYDROCHLORIDE | C01BD01 | 13 | 4,249 | 4,262 | 1.27(0.74–2.19) | 0.31(-0.46) |
| TESTOSTERONE | G03BA03 | 13 | 11,945 | 11,958 | 0.45(0.26–0.78) | -1.09(-1.86) |
| FAM-TRASTUZUMAB DERUXTECAN-NXKI | L01FD04 | 13 | 4,061 | 4,074 | 1.33(0.77–2.29) | 0.37(-0.4) |
| ICOSAPENT ETHYL | #N/A | 13 | 1,683 | 1,696 | 3.2(1.86–5.53) | 1.46(0.69) |
| GALCANEZUMAB-GNLM | N02CD02 | 13 | 9,505 | 9,518 | 0.57(0.33–0.98) | -0.77(-1.54) |
| VORICONAZOLE | J02AC03 | 13 | 5,975 | 5,988 | 0.9(0.52–1.55) | -0.14(-0.91) |
| SULFAMETHOXAZOLE\TRIMETHOPRIM | J01EE01 | 13 | 6,806 | 6,819 | 0.79(0.46–1.36) | -0.31(-1.09) |
| MOXIFLOXACIN HYDROCHLORIDE | J01MA14 | 13 | 6,416 | 6,429 | 0.84(0.49–1.45) | -0.23(-1.01) |
| ALBIGLUTIDE | A10BJ04 | 13 | 8,242 | 8,255 | 0.65(0.38–1.13) | -0.58(-1.35) |
| PIMAVANSERIN TARTRATE | N05AX17 | 13 | 37,735 | 37,748 | 0.14(0.08–0.25) | -2.71(-3.48) |
| CISPLATIN | L01XA01 | 13 | 7,456 | 7,469 | 0.72(0.42–1.25) | -0.44(-1.21) |
| LOSARTAN | C09CA01 | 12 | 3,698 | 3,710 | 1.35(0.76–2.37) | 0.39(-0.41) |
| PREDNISONE | A07EA03 | 12 | 8,109 | 8,121 | 0.61(0.35–1.08) | -0.66(-1.46) |
| GLECAPREVIR\PIBRENTASVIR | J05AP57 | 12 | 12,940 | 12,952 | 0.38(0.22–0.68) | -1.31(-2.11) |
| PANTOPRAZOLE SODIUM | A02BC02 | 12 | 8,249 | 8,261 | 0.6(0.34–1.06) | -0.68(-1.48) |
| LORAZEPAM | N05BA06 | 12 | 9,691 | 9,703 | 0.51(0.29–0.9) | -0.91(-1.71) |
| PEMETREXED DISODIUM | L01BA04 | 12 | 4,774 | 4,786 | 1.04(0.59–1.84) | 0.05(-0.75) |
| ERDAFITINIB | L01EN01 | 12 | 566 | 578 | 8.8(4.96–15.58) | 2.44(1.63) |
| FLUCONAZOLE | D01AC15 | 12 | 5,566 | 5,578 | 0.89(0.51–1.58) | -0.15(-0.95) |
| SIMEPREVIR | J05AP05 | 12 | 4,807 | 4,819 | 1.04(0.59–1.82) | 0.05(-0.76) |
| SULFASALAZINE | A07EC01 | 12 | 3,076 | 3,088 | 1.62(0.92–2.85) | 0.62(-0.18) |
| METHYLPREDNISOLONE SODIUM SUCCINATE | H02AB04 | 12 | 7,410 | 7,422 | 0.67(0.38–1.18) | -0.54(-1.34) |
| METHYLPHENIDATE HYDROCHLORIDE | N06BA04 | 12 | 6,689 | 6,701 | 0.74(0.42–1.31) | -0.4(-1.2) |
| SUVOREXANT | N05CJ01 | 12 | 5,139 | 5,151 | 0.97(0.55–1.71) | -0.04(-0.84) |
| DOCETAXEL\DOCETAXEL ANHYDROUS | #N/A | 11 | 1,811 | 1,822 | 2.52(1.39–4.56) | 1.16(0.32) |
| SUMATRIPTAN SUCCINATE | N02CC01 | 11 | 9,553 | 9,564 | 0.48(0.26–0.86) | -1.0(-1.83) |
| CANAGLIFLOZIN | A10BK02 | 11 | 17,525 | 17,536 | 0.26(0.14–0.47) | -1.85(-2.68) |
| FAMOTIDINE | A02BA03 | 11 | 1,502 | 1,513 | 3.04(1.68–5.5) | 1.37(0.53) |
| INSULIN HUMAN | A10AB01 | 11 | 5,900 | 5,911 | 0.77(0.43–1.4) | -0.34(-1.18) |
| MIRABEGRON | G04BD12 | 11 | 8,682 | 8,693 | 0.53(0.29–0.95) | -0.87(-1.7) |
| EPIRUBICIN HYDROCHLORIDE | L01DB03 | 11 | 3,095 | 3,106 | 1.47(0.82–2.66) | 0.5(-0.33) |
| SECNIDAZOLE | P01AB07 | 11 | 195 | 206 | 23.4(12.75–42.96) | 3.0(2.14) |
| NITROFURANTOIN | J01XE01 | 11 | 4,015 | 4,026 | 1.14(0.63–2.05) | 0.17(-0.67) |
| ATORVASTATIN CALCIUM | C10AA05 | 11 | 13,894 | 13,905 | 0.33(0.18–0.59) | -1.52(-2.36) |
| TACROLIMUS | D11AH01 | 11 | 23,150 | 23,161 | 0.2(0.11–0.36) | -2.24(-3.07) |
| PEXIDARTINIB HYDROCHLORIDE | L01EX15 | 11 | 1,056 | 1,067 | 4.32(2.39–7.83) | 1.75(0.91) |
| LANREOTIDE ACETATE | H01CB03 | 11 | 9,894 | 9,905 | 0.46(0.26–0.83) | -1.05(-1.88) |
| PAZOPANIB | L01EX03 | 11 | 2,054 | 2,065 | 2.22(1.23–4.02) | 1.01(0.17) |
| AZITHROMYCIN DIHYDRATE | J01FA10 | 11 | 2,750 | 2,761 | 1.66(0.92–3.0) | 0.65(-0.18) |
| PEGINTERFERON BETA-1A | L03AB13 | 11 | 14,290 | 14,301 | 0.32(0.18–0.58) | -1.56(-2.39) |
| SUMATRIPTAN | N02CC01 | 11 | 3,282 | 3,293 | 1.39(0.77–2.51) | 0.43(-0.41) |
| SIMVASTATIN | C10AA01 | 11 | 6,859 | 6,870 | 0.66(0.37–1.2) | -0.55(-1.38) |
| ALPRAZOLAM | N05BA12 | 11 | 14,930 | 14,941 | 0.31(0.17–0.55) | -1.62(-2.46) |
| DABIGATRAN ETEXILATE MESYLATE | B01AE07 | 11 | 19,265 | 19,276 | 0.24(0.13–0.43) | -1.98(-2.81) |
| TEMOZOLOMIDE | L01AX03 | 11 | 6,643 | 6,654 | 0.69(0.38–1.24) | -0.5(-1.34) |
| CASIRIVIMAB\IMDEVIMAB | J06BD07 | 10 | 10,586 | 10,596 | 0.39(0.21–0.73) | -1.27(-2.14) |
| OXYCODONE HYDROCHLORIDE | N02AA05 | 10 | 8,880 | 8,890 | 0.47(0.25–0.87) | -1.03(-1.9) |
| ROMIDEPSIN | L01XH02 | 10 | 1,139 | 1,149 | 3.64(1.95–6.79) | 1.55(0.67) |
| USTEKINUMAB | L04AC05 | 10 | 44,086 | 44,096 | 0.09(0.05–0.17) | -3.28(-4.15) |
| SIPONIMOD | L04AE03 | 10 | 9,758 | 9,768 | 0.42(0.23–0.79) | -1.16(-2.03) |
| LEVETIRACETAM | N03AX14 | 10 | 15,364 | 15,374 | 0.27(0.15–0.5) | -1.79(-2.66) |
| MINOCYCLINE HYDROCHLORIDE | J01AA08 | 10 | 1,137 | 1,147 | 3.65(1.96–6.8) | 1.55(0.67) |
| EXENATIDE | A10BJ01 | 10 | 4,196 | 4,206 | 0.99(0.53–1.84) | -0.02(-0.89) |
| DOXYCYCLINE HYCLATE | J01AA02 | 10 | 2,439 | 2,449 | 1.7(0.91–3.16) | 0.67(-0.2) |
| ECULIZUMAB | L04AJ01 | 10 | 24,492 | 24,502 | 0.17(0.09–0.31) | -2.45(-3.32) |
| EZETIMIBE | C10AX09 | 10 | 4,294 | 4,304 | 0.97(0.52–1.8) | -0.05(-0.92) |
| CEFUROXIME AXETIL | J01DC02 | 10 | 1,685 | 1,695 | 2.46(1.32–4.58) | 1.11(0.24) |
| ELTROMBOPAG OLAMINE | B02BX05 | 10 | 10,451 | 10,461 | 0.4(0.21–0.74) | -1.25(-2.12) |
| IDELALISIB | L01EM01 | 10 | 7,996 | 8,006 | 0.52(0.28–0.96) | -0.88(-1.75) |
| CLONAZEPAM | N03AE01 | 10 | 8,668 | 8,678 | 0.48(0.26–0.89) | -0.99(-1.86) |
| TOBRAMYCIN | J01GB01 | 10 | 5,128 | 5,138 | 0.81(0.43–1.5) | -0.28(-1.15) |
| BRIGATINIB | L01ED04 | 10 | 4,086 | 4,096 | 1.01(0.55–1.89) | 0.02(-0.85) |
| METHYLPREDNISOLONE | D07AA01 | 10 | 4,569 | 4,579 | 0.91(0.49–1.69) | -0.13(-1.0) |
| OXYMETAZOLINE | D11AX27 | 10 | 99 | 109 | 41.9(21.86–80.3) | 3.12(2.21) |
| CEPHALEXIN | J01DB01 | 10 | 2,673 | 2,683 | 1.55(0.83–2.89) | 0.56(-0.31) |
| CHOLESTYRAMINE | C10AC01 | 9 | 1,082 | 1,091 | 3.45(1.79–6.65) | 1.46(0.55) |
| DROXIDOPA | C01CA27 | 9 | 20,215 | 20,224 | 0.18(0.1–0.35) | -2.31(-3.22) |
| TEDUGLUTIDE | A16AX08 | 9 | 22,954 | 22,963 | 0.16(0.08–0.31) | -2.49(-3.4) |
| DAROLUTAMIDE | L02BB06 | 9 | 1,592 | 1,601 | 2.34(1.22–4.52) | 1.04(0.13) |
| STANNOUS FLUORIDE | A01AA04 | 9 | 421 | 430 | 8.87(4.58–17.16) | 2.3(1.37) |
| GEMCITABINE | L01BC05 | 9 | 3,830 | 3,839 | 0.97(0.51–1.87) | -0.03(-0.95) |
| CELECOXIB | L01XX33 | 9 | 7,376 | 7,385 | 0.51(0.26–0.97) | -0.91(-1.82) |
| ITRACONAZOLE | J02AC02 | 9 | 1,346 | 1,355 | 2.77(1.44–5.34) | 1.23(0.31) |
| CLADRIBINE | L01BB04 | 9 | 6,223 | 6,232 | 0.6(0.31–1.15) | -0.68(-1.59) |
| IOPROMIDE | V08AB05 | 9 | 7,721 | 7,730 | 0.48(0.25–0.93) | -0.97(-1.88) |
| TAMSULOSIN | G04CA02 | 9 | 3,319 | 3,328 | 1.12(0.58–2.16) | 0.15(-0.76) |
| ENCORAFENIB | L01EC03 | 9 | 5,120 | 5,129 | 0.73(0.38–1.4) | -0.42(-1.33) |
| DEXLANSOPRAZOLE | A02BC06 | 9 | 2,012 | 2,021 | 1.86(0.96–3.57) | 0.77(-0.15) |
| SOMATROPIN | H01AC01 | 9 | 33,043 | 33,052 | 0.11(0.06–0.22) | -3.01(-3.92) |
| SOFOSBUVIR | J05AP08 | 9 | 7,366 | 7,375 | 0.51(0.26–0.97) | -0.91(-1.82) |
| BEPOTASTINE BESILATE | #N/A | 9 | 90 | 99 | 41.48(20.9–82.31) | 3.01(2.05) |
| ELEXACAFTOR\IVACAFTOR\TEZACAFTOR | R07AX32 | 9 | 7,881 | 7,890 | 0.47(0.25–0.91) | -1.0(-1.91) |
| ALLOPURINOL | M04AA01 | 9 | 6,558 | 6,567 | 0.57(0.3–1.09) | -0.75(-1.66) |
| FEBUXOSTAT | M04AA03 | 9 | 2,420 | 2,429 | 1.54(0.8–2.97) | 0.55(-0.37) |
| IRBESARTAN | C09CA04 | 9 | 2,332 | 2,341 | 1.6(0.83–3.08) | 0.59(-0.32) |
| OMBITASVIR\PARITAPREVIR\RITONAVIR | J05AP53 | 9 | 9,940 | 9,949 | 0.38(0.2–0.72) | -1.32(-2.23) |
| TRAMADOL | N02AX02 | 9 | 7,960 | 7,969 | 0.47(0.24–0.9) | -1.01(-1.93) |
| .ALPHA.1-PROTEINASE INHIBITOR HUMAN | #N/A | 9 | 10,708 | 10,717 | 0.35(0.18–0.67) | -1.42(-2.33) |
| PARATHYROID HORMONE | H05AA03 | 9 | 6,974 | 6,983 | 0.53(0.28–1.03) | -0.83(-1.75) |
| PATIROMER | #N/A | 9 | 9,583 | 9,592 | 0.39(0.2–0.75) | -1.27(-2.18) |
| ZAVEGEPANT HYDROCHLORIDE | N02CD08 | 9 | 84 | 93 | 44.44(22.35–88.39) | 3.03(2.07) |
| BOSUTINIB MONOHYDRATE | L01EA04 | 9 | 4,451 | 4,460 | 0.84(0.44–1.61) | -0.23(-1.14) |
| IMATINIB | L01EA01 | 9 | 3,568 | 3,577 | 1.05(0.54–2.01) | 0.06(-0.86) |
| ERIBULIN MESYLATE | L01XX41 | 9 | 3,035 | 3,044 | 1.23(0.64–2.37) | 0.26(-0.65) |
| ENASIDENIB MESYLATE | L01XM01 | 8 | 1,331 | 1,339 | 2.49(1.24–5.0) | 1.09(0.13) |
| VORINOSTAT | L01XH01 | 8 | 550 | 558 | 6.03(3.0–12.13) | 1.94(0.97) |
| ACETAMINOPHEN\CHLORPHENIRAMINE MALEATE\DEXTROMETHORPHAN HYDROBROMIDE\DOXYLAMINE SUCCINATE\PHENYLEPHRINE HYDROCHLORIDE | #N/A | 8 | 8 | 16 | 414.79(155.66–1,105.27) | 3.11(1.92) |
| ARTICAINE HYDROCHLORIDE\EPINEPHRINE BITARTRATE | #N/A | 8 | 1,130 | 1,138 | 2.94(1.46–5.89) | 1.27(0.3) |
| IRON SUCROSE | B03AB02 | 8 | 5,302 | 5,310 | 0.63(0.31–1.25) | -0.61(-1.58) |
| METHOTREXATE | L01BA01 | 8 | 21,972 | 21,980 | 0.15(0.08–0.3) | -2.58(-3.54) |
| LINACLOTIDE | A06AX04 | 8 | 4,451 | 4,459 | 0.75(0.37–1.49) | -0.38(-1.35) |
| CARFILZOMIB | L01XG02 | 8 | 9,374 | 9,382 | 0.35(0.18–0.71) | -1.39(-2.35) |
| TIOTROPIUM BROMIDE | R03BB04 | 8 | 2,299 | 2,307 | 1.44(0.72–2.89) | 0.46(-0.51) |
| GEMCITABINE HYDROCHLORIDE | L01BC05 | 8 | 7,227 | 7,235 | 0.46(0.23–0.92) | -1.03(-2.0) |
| EPOPROSTENOL | B01AC09 | 8 | 13,947 | 13,955 | 0.24(0.12–0.48) | -1.94(-2.9) |
| RADIUM RA-223 DICHLORIDE | #N/A | 8 | 3,744 | 3,752 | 0.89(0.44–1.77) | -0.16(-1.12) |
| ELACESTRANT | L02BA04 | 8 | 3,668 | 3,676 | 0.9(0.45–1.81) | -0.13(-1.09) |
| RIFAXIMIN | A07AA11 | 8 | 6,187 | 6,195 | 0.54(0.27–1.07) | -0.82(-1.78) |
| LISDEXAMFETAMINE DIMESYLATE | N06BA12 | 8 | 5,894 | 5,902 | 0.56(0.28–1.13) | -0.76(-1.72) |
| SULFUR HEXAFLUORIDE | V08DA05 | 8 | 3,427 | 3,435 | 0.97(0.48–1.94) | -0.04(-1.01) |
| TAZEMETOSTAT HYDROBROMIDE | L01XX72 | 8 | 567 | 575 | 5.85(2.91–11.76) | 1.92(0.95) |
| SODIUM CHLORIDE | A12CA01 | 8 | 5,967 | 5,975 | 0.56(0.28–1.11) | -0.77(-1.74) |
| SPIRONOLACTONE | C03DA01 | 8 | 4,334 | 4,342 | 0.77(0.38–1.53) | -0.35(-1.31) |
| CENEGERMIN-BKBJ | S01XA24 | 8 | 7,381 | 7,389 | 0.45(0.22–0.9) | -1.06(-2.02) |
| FLUOXETINE HYDROCHLORIDE | N06AB03 | 8 | 11,101 | 11,109 | 0.3(0.15–0.6) | -1.62(-2.59) |
| AZITHROMYCIN | J01FA10 | 8 | 4,000 | 4,008 | 0.83(0.41–1.66) | -0.24(-1.21) |
| BIMATOPROST | S01EE03 | 8 | 3,808 | 3,816 | 0.87(0.44–1.74) | -0.18(-1.14) |
| ALECTINIB | L01ED03 | 8 | 2,333 | 2,341 | 1.42(0.71–2.85) | 0.44(-0.52) |
| TRAMETINIB DIMETHYL SULFOXIDE | L01EE01 | 8 | 6,183 | 6,191 | 0.54(0.27–1.07) | -0.82(-1.78) |
| VALSARTAN | C09CA03 | 8 | 6,688 | 6,696 | 0.5(0.25–0.99) | -0.93(-1.89) |
| HOMEOPATHICS\ZINC ACETATE ANHYDROUS\ZINC GLUCONATE | #N/A | 8 | 7 | 15 | 474.04(171.88–1,307.37) | 3.12(1.91) |
| THALIDOMIDE | L04AX02 | 8 | 6,665 | 6,673 | 0.5(0.25–1.0) | -0.92(-1.89) |
| ABOBOTULINUMTOXINA | #N/A | 8 | 7,095 | 7,103 | 0.47(0.23–0.94) | -1.01(-1.97) |
| ACETAMINOPHEN\ASPIRIN\CAFFEINE | #N/A | 8 | 1,112 | 1,120 | 2.98(1.49–5.98) | 1.28(0.32) |
| DIROXIMEL FUMARATE | L04AX09 | 8 | 7,917 | 7,925 | 0.42(0.21–0.84) | -1.16(-2.12) |
| ROMOSOZUMAB-AQQG | M05BX06 | 8 | 4,071 | 4,079 | 0.81(0.41–1.63) | -0.27(-1.23) |
| LORATADINE\PSEUDOEPHEDRINE SULFATE | #N/A | 8 | 1,976 | 1,984 | 1.68(0.84–3.36) | 0.64(-0.32) |
| BEBTELOVIMAB | #N/A | 8 | 3,048 | 3,056 | 1.09(0.54–2.18) | 0.11(-0.86) |
| ESTRADIOL | G03CA03 | 8 | 12,150 | 12,158 | 0.27(0.14–0.55) | -1.75(-2.71) |
| BRIMONIDINE TARTRATE\BRINZOLAMIDE | S01EC54 | 7 | 799 | 806 | 3.63(1.73–7.65) | 1.44(0.42) |
| CANAKINUMAB | L04AC08 | 7 | 11,272 | 11,279 | 0.26(0.12–0.54) | -1.81(-2.84) |
| TRAMADOL HYDROCHLORIDE | N02AX02 | 7 | 6,447 | 6,454 | 0.45(0.21–0.94) | -1.05(-2.07) |
| RAMUCIRUMAB | L01FG02 | 7 | 3,791 | 3,798 | 0.77(0.36–1.61) | -0.34(-1.36) |
| LOPERAMIDE HYDROCHLORIDE | A07DA03 | 7 | 2,841 | 2,848 | 1.02(0.49–2.15) | 0.03(-1.0) |
| ACYCLOVIR | J05AB01 | 7 | 4,144 | 4,151 | 0.7(0.33–1.47) | -0.46(-1.48) |
| CLOFAZIMINE | J04BA01 | 7 | 3,624 | 3,631 | 0.8(0.38–1.68) | -0.28(-1.31) |
| VILAZODONE HYDROCHLORIDE | N06AX24 | 7 | 1,395 | 1,402 | 2.08(0.99–4.37) | 0.87(-0.15) |
| OFLOXACIN | J01MA01 | 7 | 1,608 | 1,615 | 1.81(0.86–3.79) | 0.71(-0.31) |
| OMEPRAZOLE MAGNESIUM | A02BC01 | 7 | 1,455 | 1,462 | 2.0(0.95–4.19) | 0.82(-0.2) |
| GILTERITINIB | L01EX13 | 7 | 4,168 | 4,175 | 0.7(0.33–1.46) | -0.47(-1.49) |
| ACETAMINOPHEN\CHLORPHENIRAMINE MALEATE\DEXTROMETHORPHAN HYDROBROMIDE\PHENYLEPHRINE HYDROCHLORIDE OR ACETAMINOPHEN\DEXTROMETHORPHAN HYDROBROMIDE\DOXYLAMINE SUCCINATE\PHENYLEPHRINE HYDROCHLORIDE | #N/A | 7 | 20 | 27 | 145.17(61.38–343.35) | 2.91(1.75) |
| CEFTRIAXONE SODIUM | J01DD04 | 7 | 2,355 | 2,362 | 1.23(0.59–2.59) | 0.26(-0.76) |
| ATOMOXETINE HYDROCHLORIDE | N06BA09 | 7 | 3,742 | 3,749 | 0.78(0.37–1.63) | -0.33(-1.35) |
| DARATUMUMAB | L01FC01 | 7 | 11,026 | 11,033 | 0.26(0.13–0.55) | -1.78(-2.8) |
| TIOPRONIN | G04BX16 | 7 | 334 | 341 | 8.69(4.11–18.38) | 2.13(1.1) |
| DORZOLAMIDE HYDROCHLORIDE | S01EC03 | 7 | 277 | 284 | 10.48(4.95–22.19) | 2.25(1.21) |
| ICATIBANT ACETATE | B06AC02 | 7 | 7,302 | 7,309 | 0.4(0.19–0.83) | -1.22(-2.24) |
| ASCORBIC ACID\POLYETHYLENE GLYCOL 3350\POTASSIUM CHLORIDE\SODIUM ASCORBATE\SODIUM CHLORIDE\SODIUM SULFATE | #N/A | 7 | 2,750 | 2,757 | 1.06(0.5–2.22) | 0.07(-0.95) |
| PACRITINIB | L01EJ03 | 7 | 867 | 874 | 3.35(1.59–7.05) | 1.37(0.34) |
| TALQUETAMAB-TGVS | L01FX29 | 7 | 131 | 138 | 22.16(10.36–47.41) | 2.58(1.53) |
| DABRAFENIB | L01EC02 | 7 | 4,615 | 4,622 | 0.63(0.3–1.32) | -0.6(-1.62) |
| TAFAMIDIS | N07XX08 | 7 | 2,901 | 2,908 | 1.0(0.48–2.1) | 0.0(-1.02) |
| MOXIFLOXACIN | J01MA14 | 7 | 2,431 | 2,438 | 1.19(0.57–2.51) | 0.22(-0.8) |
| MEROPENEM | J01DH02 | 7 | 2,406 | 2,413 | 1.21(0.57–2.53) | 0.23(-0.79) |
| ANAKINRA | L04AC03 | 7 | 10,563 | 10,570 | 0.27(0.13–0.58) | -1.72(-2.74) |
| PANCRELIPASE AMYLASE\PANCRELIPASE LIPASE\PANCRELIPASE PROTEASE | #N/A | 7 | 4,358 | 4,365 | 0.67(0.32–1.4) | -0.52(-1.55) |
| SITAGLIPTIN PHOSPHATE | A10BH01 | 7 | 5,081 | 5,088 | 0.57(0.27–1.2) | -0.73(-1.75) |
| LIDOCAINE | C01BB01 | 7 | 6,178 | 6,185 | 0.47(0.22–0.99) | -0.99(-2.01) |
| DULOXETINE | N06AX21 | 7 | 5,880 | 5,887 | 0.49(0.24–1.04) | -0.92(-1.94) |
| FLUTICASONE FUROATEUMECLIDINIUM BROMIDEVILANTEROL TRIFENATATE | #N/A | 6 | 623 | 629 | 3.99(1.79–8.93) | 1.48(0.38) |
| CALCIUM CARBONATE\FAMOTIDINE\MAGNESIUM HYDROXIDE | A02BA53 | 6 | 278 | 284 | 8.95(3.99–20.1) | 2.05(0.95) |
| NAPROXEN | G02CC02 | 6 | 6,209 | 6,215 | 0.4(0.18–0.89) | -1.19(-2.28) |
| PRASUGREL HYDROCHLORIDE | B01AC22 | 6 | 3,191 | 3,197 | 0.78(0.35–1.74) | -0.31(-1.4) |
| RIMEGEPANT SULFATE | N02CD06 | 6 | 1,937 | 1,943 | 1.28(0.58–2.86) | 0.3(-0.79) |
| INTERFERON ALFA-2B | L03AB05 | 6 | 950 | 956 | 2.62(1.17–5.85) | 1.08(-0.01) |
| MESALAMINE | A07EC02 | 6 | 4,121 | 4,127 | 0.6(0.27–1.34) | -0.64(-1.73) |
| BICTEGRAVIR SODIUM\EMTRICITABINE\TENOFOVIR ALAFENAMIDE FUMARATE | J05AR20 | 6 | 2,258 | 2,264 | 1.1(0.49–2.46) | 0.12(-0.97) |
| ALCOHOL | D08AX08 | 6 | 1,294 | 1,300 | 1.92(0.86–4.29) | 0.76(-0.33) |
| CARBAMAZEPINE | N03AF01 | 6 | 7,898 | 7,904 | 0.31(0.14–0.7) | -1.52(-2.61) |
| CLARITHROMYCIN CITRATE | #N/A | 6 | 90 | 96 | 27.65(12.1–63.19) | 2.5(1.38) |
| DOFETILIDE | C01BD04 | 6 | 2,605 | 2,611 | 0.96(0.43–2.13) | -0.06(-1.15) |
| LEVOTHYROXINE | #N/A | 6 | 2,942 | 2,948 | 0.85(0.38–1.88) | -0.21(-1.3) |
| CEDAZURIDINE\DECITABINE | L01BC58 | 6 | 2,211 | 2,217 | 1.13(0.5–2.51) | 0.14(-0.95) |
| AZACITIDINE | L01BC07 | 6 | 12,406 | 12,412 | 0.20(0.09–0.45) | -2.14(-3.23) |
| CLINDAMYCIN\CLINDAMYCIN PHOSPHATE | J01FF01 | 6 | 834 | 840 | 2.98(1.34–6.66) | 1.21(0.12) |
| PAROXETINE HYDROCHLORIDE | N06AB05 | 6 | 4,531 | 4,537 | 0.55(0.25–1.22) | -0.77(-1.86) |
| EPTINEZUMAB-JJMR | N02CD05 | 6 | 3,316 | 3,322 | 0.75(0.34–1.67) | -0.36(-1.45) |
| DORZOLAMIDE HYDROCHLORIDE\TIMOLOL MALEATE | S01ED51 | 6 | 398 | 404 | 6.25(2.79–14.0) | 1.83(0.73) |
| DURVALUMAB | L01FF03 | 6 | 5,576 | 5,582 | 0.45(0.2–0.99) | -1.04(-2.14) |
| ILOPROST | B01AC11 | 6 | 5,891 | 5,897 | 0.42(0.19–0.94) | -1.12(-2.21) |
| FORMOTEROL FUMARATE DIHYDRATE\MOMETASONE FUROATE | #N/A | 6 | 2,056 | 2,062 | 1.21(0.54–2.7) | 0.23(-0.86) |
| AMIODARONE | C01BD01 | 6 | 4,295 | 4,301 | 0.58(0.26–1.29) | -0.7(-1.79) |
| ASENAPINE MALEATE | N05AH05 | 6 | 976 | 982 | 2.55(1.14–5.69) | 1.06(-0.04) |
| BALOXAVIR MARBOXIL | J05AX25 | 6 | 1,271 | 1,277 | 1.96(0.88–4.37) | 0.78(-0.31) |
| PEXIDARTINIB | L01EX15 | 6 | 525 | 531 | 4.74(2.12–10.6) | 1.62(0.52) |
| ARMODAFINIL | N06BA13 | 6 | 927 | 933 | 2.68(1.2–5.99) | 1.11(0.01) |
| HUMAN C1-ESTERASE INHIBITOR | #N/A | 6 | 11,763 | 11,769 | 0.21(0.09–0.47) | -2.07(-3.16) |
| METOPROLOL SUCCINATE | C07AB02 | 6 | 3,175 | 3,181 | 0.78(0.35–1.75) | -0.31(-1.4) |
| GADOBENATE DIMEGLUMINE | V08CA08 | 6 | 3,257 | 3,263 | 0.76(0.34–1.7) | -0.34(-1.43) |
| INSULIN DETEMIR | A10AE05 | 6 | 2,498 | 2,504 | 1.0(0.45–2.22) | -0.01(-1.1) |
| FESOTERODINE FUMARATE | G04BD11 | 6 | 1,237 | 1,243 | 2.01(0.9–4.49) | 0.81(-0.28) |
| ROMIPLOSTIM | B02BX04 | 6 | 4,206 | 4,212 | 0.59(0.27–1.32) | -0.67(-1.76) |
| PONATINIB | L01EA05 | 6 | 11,722 | 11,728 | 0.21(0.10–0.47) | -2.06(-3.15) |
| DAPAGLIFLOZIN | A10BK01 | 6 | 6,357 | 6,363 | 0.39(0.18–0.87) | -1.22(-2.31) |
| ZOLPIDEM TARTRATE | N05CF02 | 6 | 6,447 | 6,453 | 0.39(0.17–0.86) | -1.24(-2.33) |
| CALCIUM OXYBATE\MAGNESIUM OXYBATE\POTASSIUM OXYBATE\SODIUM OXYBATE | #N/A | 6 | 4,079 | 4,085 | 0.61(0.27–1.36) | -0.63(-1.72) |
| BENZONATATE | R05DB01 | 6 | 488 | 494 | 5.1(2.28–11.41) | 1.68(0.58) |
| FENOFIBRATE | C10AB05 | 6 | 1,616 | 1,622 | 1.54(0.69–3.43) | 0.51(-0.58) |
| OLODATEROL HYDROCHLORIDE\TIOTROPIUM BROMIDE MONOHYDRATE | #N/A | 6 | 2,502 | 2,508 | 0.99(0.45–2.22) | -0.01(-1.1) |
| TELOTRISTAT ETHYL | A16AX15 | 6 | 3,443 | 3,449 | 0.72(0.32–1.61) | -0.41(-1.5) |
| GLYCEROL PHENYLBUTYRATE | A16AX09 | 6 | 1,117 | 1,123 | 2.23(1.0–4.97) | 0.92(-0.18) |
| SOLIFENACIN SUCCINATE | G04BD08 | 6 | 2,230 | 2,236 | 1.12(0.5–2.49) | 0.13(-0.96) |
| MITOTANE | L01XX23 | 6 | 1,005 | 1,011 | 2.48(1.11–5.52) | 1.03(-0.07) |
| LOMITAPIDE MESYLATE | C10AX12 | 6 | 4,070 | 4,076 | 0.61(0.27–1.36) | -0.63(-1.72) |
| CERITINIB | L01ED02 | 6 | 4,855 | 4,861 | 0.51(0.23–1.14) | -0.86(-1.95) |
| DORZOLAMIDE | S01EC03 | 6 | 124 | 130 | 20.07(8.84–45.54) | 2.41(1.29) |
| PAROXETINE | N06AB05 | 6 | 6,968 | 6,974 | 0.36(0.16–0.79) | -1.34(-2.44) |
| FRUQUINTINIB | L01EK04 | 6 | 1,831 | 1,837 | 1.36(0.61–3.03) | 0.37(-0.72) |
| VALACYCLOVIR HYDROCHLORIDE | J05AB11 | 6 | 6,295 | 6,301 | 0.4(0.18–0.88) | -1.21(-2.3) |
| MONTELUKAST SODIUM | R03DC03 | 6 | 16,768 | 16,774 | 0.15(0.07–0.33) | -2.56(-3.65) |
| CEFTRIAXONE | J01DD04 | 5 | 2,386 | 2,391 | 0.87(0.36–2.09) | -0.17(-1.35) |
| DEOXYCHOLIC ACID | D11AX24 | 5 | 1,475 | 1,480 | 1.41(0.58–3.38) | 0.39(-0.79) |
| CLOZAPINE | N05AH02 | 5 | 50,759 | 50,764 | 0.04(0.02–0.1) | -4.36(-5.54) |
| FEXOFENADINE\FEXOFENADINE HYDROCHLORIDE | R06AX26 | 5 | 1,105 | 1,110 | 1.88(0.78–4.52) | 0.71(-0.47) |
| ETANERCEPT-SZZS | #N/A | 5 | 5,504 | 5,509 | 0.38(0.16–0.91) | -1.25(-2.43) |
| VANCOMYCIN | A07AA09 | 5 | 7,693 | 7,698 | 0.27(0.11–0.65) | -1.7(-2.88) |
| LAPATINIB DITOSYLATE | L01EH01 | 5 | 3,148 | 3,153 | 0.66(0.27–1.58) | -0.52(-1.7) |
| TALQUETAMAB | L01FX29 | 5 | 218 | 223 | 9.51(3.92–23.09) | 1.96(0.77) |
| EXEMESTANE | L02BG06 | 5 | 4,290 | 4,295 | 0.48(0.2–1.16) | -0.92(-2.1) |
| GADOTERIDOL | V08CA04 | 5 | 1,361 | 1,366 | 1.52(0.63–3.67) | 0.48(-0.7) |
| NYSTATIN | A07AA02 | 5 | 431 | 436 | 4.81(1.99–11.62) | 1.55(0.36) |
| GUSELKUMAB | L04AC16 | 5 | 11,515 | 11,520 | 0.18(0.07–0.43) | -2.26(-3.44) |
| TICAGRELOR | B01AC24 | 5 | 8,060 | 8,065 | 0.26(0.11–0.62) | -1.77(-2.94) |
| TOLVAPTAN | C03XA01 | 5 | 8,898 | 8,903 | 0.23(0.1–0.56) | -1.9(-3.08) |
| POTASSIUM NITRATE\SODIUM FLUORIDE | #N/A | 5 | 268 | 273 | 7.74(3.19–18.74) | 1.85(0.66) |
| HYDROXYPROGESTERONE CAPROATE | G03DA03 | 5 | 15,157 | 15,162 | 0.14(0.06–0.33) | -2.64(-3.82) |
| METHOXY POLYETHYLENE GLYCOL-EPOETIN BETA | B03XA03 | 5 | 8,444 | 8,449 | 0.25(0.1–0.59) | -1.83(-3.01) |
| BUSPIRONE HYDROCHLORIDE | N05BE01 | 5 | 898 | 903 | 2.31(0.96–5.56) | 0.92(-0.26) |
| BRENTUXIMAB VEDOTIN | L01FX05 | 5 | 7,900 | 7,905 | 0.26(0.11–0.63) | -1.74(-2.92) |
| AMOXICILLIN\CLARITHROMYCIN\LANSOPRAZOLE | J01RA01 | 5 | 138 | 143 | 15.03(6.16–36.68) | 2.16(0.95) |
| DACLATASVIR | J05AP07 | 5 | 1,926 | 1,931 | 1.08(0.45–2.59) | 0.09(-1.09) |
| MELOXICAM | M01AC06 | 5 | 2,116 | 2,121 | 0.98(0.41–2.36) | -0.03(-1.2) |
| OXYBUTYNIN | G04BD04 | 5 | 1,392 | 1,397 | 1.49(0.62–3.58) | 0.46(-0.72) |
| HUMAN IMMUNOGLOBULIN G\HYALURONIDASE RECOMBINANT HUMAN | #N/A | 5 | 3,822 | 3,827 | 0.54(0.23–1.3) | -0.77(-1.95) |
| AXICABTAGENE CILOLEUCEL | L01XL03 | 5 | 13,331 | 13,336 | 0.16(0.06–0.37) | -2.46(-3.64) |
| CABAZITAXEL | L01CD04 | 5 | 1,961 | 1,966 | 1.06(0.44–2.54) | 0.07(-1.11) |
| ACETAMINOPHEN\HYDROCODONE BITARTRATE | N02AJ22 | 5 | 2,039 | 2,044 | 1.02(0.42–2.45) | 0.02(-1.16) |
| BUPROPION | N06AX12 | 5 | 2,563 | 2,568 | 0.81(0.34–1.95) | -0.26(-1.44) |
| GEMCITABINE\GEMCITABINE HYDROCHLORIDE | L01BC05 | 5 | 2,563 | 2,568 | 0.81(0.34–1.95) | -0.26(-1.44) |
| EMTRICITABINE\TENOFOVIR DISOPROXIL FUMARATE | #N/A | 5 | 19,765 | 19,770 | 0.10(0.04–0.25) | -3.02(-4.2) |
| DICLOFENAC | D11AX18 | 5 | 5,599 | 5,604 | 0.37(0.15–0.89) | -1.27(-2.45) |
| EPINEPHRINE BITARTRATE\LIDOCAINE HYDROCHLORIDE | #N/A | 5 | 625 | 630 | 3.32(1.38–8.0) | 1.25(0.07) |
| KETOROLAC TROMETHAMINE | M01AB15 | 5 | 1,022 | 1,027 | 2.03(0.84–4.89) | 0.79(-0.39) |
| FOSTAMATINIB | B02BX09 | 5 | 2,153 | 2,158 | 0.96(0.4–2.32) | -0.05(-1.23) |
| FINGOLIMOD | L04AE01 | 5 | 1,528 | 1,533 | 1.36(0.56–3.27) | 0.36(-0.83) |
| RITUXIMAB-PVVR | L01FA01 | 5 | 9,247 | 9,252 | 0.22(0.09–0.54) | -1.95(-3.13) |
| METRONIDAZOLE BENZOATE | J01XD01 | 5 | 159 | 164 | 13.04(5.35–31.77) | 2.1(0.9) |
| EMPAGLIFLOZIN\METFORMIN HYDROCHLORIDE | #N/A | 5 | 1,398 | 1,403 | 1.48(0.62–3.57) | 0.45(-0.73) |
| QUETIAPINE | N05AH04 | 5 | 18,875 | 18,880 | 0.11(0.05–0.26) | -2.95(-4.13) |
| CINACALCET HYDROCHLORIDE | H05BX01 | 5 | 3,087 | 3,092 | 0.67(0.28–1.61) | -0.49(-1.67) |
| LINAGLIPTIN | A10BH05 | 5 | 4,321 | 4,326 | 0.48(0.2–1.15) | -0.93(-2.11) |
| ZONISAMIDE | N03AX15 | 5 | 814 | 819 | 2.55(1.06–6.14) | 1.01(-0.17) |
| IOHEXOL | V08AB02 | 5 | 6,571 | 6,576 | 0.32(0.13–0.76) | -1.49(-2.67) |
| METHOTREXATE SODIUM | L04AX03 | 5 | 16,080 | 16,085 | 0.13(0.05–0.31) | -2.73(-3.9) |
| BISMUTH SUBCITRATE POTASSIUM\METRONIDAZOLE\TETRACYCLINE HYDROCHLORIDE | #N/A | 5 | 801 | 806 | 2.59(1.07–6.24) | 1.03(-0.15) |
| CIPROFLOXACIN HYDROCHLORIDE\DEXAMETHASONE | S02CA06 | 5 | 353 | 358 | 5.87(2.43–14.2) | 1.69(0.5) |
| AMITRIPTYLINE | N06AA09 | 5 | 1,864 | 1,869 | 1.11(0.46–2.68) | 0.13(-1.05) |
| ELAGOLIX SODIUM | H01CC03 | 5 | 1,329 | 1,334 | 1.56(0.65–3.75) | 0.51(-0.67) |
| INSULIN ASPART | A10AB05 | 5 | 5,728 | 5,733 | 0.36(0.15–0.87) | -1.3(-2.48) |
| CROMOLYN SODIUM | R03BC01 | 5 | 296 | 301 | 7.01(2.89–16.96) | 1.8(0.61) |
| METRONIDAZOLE\METRONIDAZOLE HYDROCHLORIDE | J01XD01 | 5 | 613 | 618 | 3.38(1.4–8.16) | 1.27(0.09) |
| BUPIVACAINE | N01BB01 | 5 | 1,082 | 1,087 | 1.92(0.8–4.61) | 0.73(-0.45) |
| LURASIDONE HYDROCHLORIDE | N05AE05 | 5 | 3,870 | 3,875 | 0.54(0.22–1.29) | -0.78(-1.96) |
| TISAGENLECLEUCEL | L01XL04 | 5 | 9,055 | 9,060 | 0.23(0.1–0.55) | -1.93(-3.1) |
| ACETAMINOPHEN\OXYCODONE HYDROCHLORIDE | #N/A | 5 | 2,858 | 2,863 | 0.73(0.3–1.74) | -0.4(-1.57) |
| BUPRENORPHINE HYDROCHLORIDE | N02AE01 | 5 | 1,623 | 1,628 | 1.28(0.53–3.07) | 0.29(-0.89) |
| FLUOROMETHOLONE | C05AA06 | 5 | 193 | 198 | 10.74(4.42–26.11) | 2.02(0.82) |

ATC, anatomical therapeutic chemical; ROR, reporting odds ratio; IC, information component; CI, confidence interval

Table S2. RORs and ICs of drugs associated with taste disorders in both the JADER database.

| **Drug** | **Cases** | **Non-cases** | **Total** | **ROR (95%CI)** | **IC (IC_025_)** |
| --- | --- | --- | --- | --- | --- |
| Total | 1,140 | 1,727,239 | 1,728,379 |  |  |
| Coronavirus RNA Vaccine | 77 | 88,295 | 88,372 | 1.34 (1.07–1.69) | 0.39(0.06) |
| Ribavirin | 42 | 26,465 | 26,507 | 2.46 (1.81–3.35) | 1.22(0.77) |
| Peginterferon alfa-2b | 31 | 16,065 | 16,096 | 2.98 (2.08–4.26) | 1.46(0.94) |
| Vorinostat | 30 | 974 | 1,004 | 47.90 (33.15–69.21) | 4.22(3.69) |
| Fluorouracil | 27 | 24,905 | 24,932 | 1.66 (1.13–2.43) | 0.68(0.13) |
| Sunitinib malate | 23 | 9,685 | 9,708 | 3.65 (2.42–5.52) | 1.70(1.10) |
| Enfortumab Vedotin | 23 | 2,276 | 2,299 | 15.61 (10.31–23.63) | 3.25(2.65) |
| Oxaliplatin | 20 | 37,993 | 38,013 | 0.79 (0.51–1.24) | -0.31(-0.95) |
| Telaprevir | 20 | 9,388 | 9,408 | 3.27 (2.10–5.09) | 1.54(0.91) |
| Nivolumab | 20 | 27,290 | 27,310 | 1.11 (0.71–1.73) | 0.14(-0.49) |
| Calcium levofolinate | 16 | 17,720 | 17,736 | 1.37 (0.84–2.25) | 0.42(-0.29) |
| Crizotinib | 16 | 2,172 | 2,188 | 11.31 (6.89–18.55) | 2.80(2.09) |
| Clarithromycin | 16 | 3,167 | 3,183 | 7.75 (4.73–12.71) | 2.45(1.75) |
| Varenicline tartrate | 15 | 2,038 | 2,053 | 11.29 (6.77–18.82) | 2.76(2.03) |
| Tegafur, gimeracil and oteracil potassium | 14 | 15,351 | 15,365 | 1.39 (0.82–2.35) | 0.43(-0.32) |
| Prednisolone | 13 | 24,722 | 24,735 | 0.79 (0.46–1.37) | -0.31(-1.08) |
| Nirmatrelvir and ritonavir | 12 | 736 | 748 | 24.96 (14.07–44.28) | 3.12(2.31) |
| Pembrolizumab | 12 | 20,141 | 20,153 | 0.90 (0.51–1.59) | -0.14(-0.94) |
| Ipilimumab | 11 | 15,304 | 15,315 | 1.09 (0.60–1.97) | 0.11(-0.73) |
| Gemcitabine hydrochloride | 10 | 8,310 | 8,320 | 1.83 (0.98–3.41) | 0.76(-0.11) |
| Dexamethasone | 10 | 19,417 | 19,427 | 0.78 (0.42–1.45) | -0.33(-1.20) |
| Cisplatin | 10 | 15,210 | 15,220 | 1.00 (0.53–1.86) | -0.01(-0.88) |
| Enzalutamide | 10 | 1,932 | 1,942 | 7.90 (4.23–14.75) | 2.27(1.39) |
| Amlodipine besylate | 10 | 2,452 | 2,462 | 6.22 (3.34–11.62) | 2.07(1.19) |
| Paclitaxel | 9 | 14,477 | 14,486 | 0.94 (0.49–1.81) | -0.08(-1.00) |
| Linezolid | 9 | 2,463 | 2,472 | 5.57 (2.89–10.75) | 1.93(1.01) |
| Bevacizumab | 9 | 25,662 | 25,671 | 0.53 (0.27–1.02) | -0.84(-1.76) |
| Azithromycin hydrate | 9 | 1,998 | 2,007 | 6.87 (3.56–13.26) | 2.10(1.19) |
| Terbinafine hydrochloride | 9 | 1,545 | 1,554 | 8.89 (4.60–17.16) | 2.30(1.38) |
| Levofloxacin | 9 | 5,088 | 5,097 | 2.69 (1.40–5.19) | 1.20(0.28) |
| Recombinant adsorbed quadrivalent human papillomavirus virus-like particle vaccine | 8 | 2,895 | 2,903 | 4.21 (2.10–8.45) | 1.63(0.66) |
| Simeprevir sodium | 8 | 5,009 | 5,017 | 2.43 (1.21–4.87) | 1.06(0.09) |
| Lenalidomide hydrate | 8 | 18,222 | 18,230 | 0.66 (0.33–1.33) | -0.53(-1.50) |
| Gefapixant citrate | 7 | 15 | 22 | 711.42 (289.51–1,748.16) | 2.98(1.79) |
| Panitumumab | 7 | 3,220 | 3,227 | 3.31 (1.57–6.96) | 1.35(0.33) |
| Tegafur and uracil | 7 | 2,377 | 2,384 | 4.48 (2.13–9.44) | 1.64(0.61) |
| Capecitabine | 7 | 9,469 | 9,476 | 1.12 (0.53–2.36) | 0.14(-0.88) |
| Laninamivir | 6 | 371 | 377 | 24.63 (10.97–55.29) | 2.49(1.38) |
| Osimertinib mesylate | 6 | 3,996 | 4,002 | 2.28 (1.02–5.09) | 0.94(-0.15) |
| Carboplatin | 6 | 13,043 | 13,049 | 0.70 (0.31–1.55) | -0.46(-1.55) |
| Recombinant adsorbed bivalent human papillomavirus-like particle vaccine | 6 | 6,040 | 6,046 | 1.51 (0.68–3.36) | 0.49(-0.61) |
| Doxorubicin hydrochloride | 6 | 8,910 | 8,916 | 1.02 (0.46–2.28) | 0.02(-1.07) |
| Palbociclib | 5 | 2,437 | 2,442 | 3.12 (1.29–7.51) | 1.20(0.02) |
| Methotrexate | 5 | 17,282 | 17,287 | 0.44 (0.18–1.05) | -1.05(-2.23) |
| Eribulin mesylate | 5 | 1,847 | 1,852 | 4.12 (1.71–9.92) | 1.43(0.25) |
| Paracetamol | 5 | 4,074 | 4,079 | 1.86 (0.77–4.49) | 0.70(-0.48) |
| Oseltamivir phosphate | 5 | 3,101 | 3,106 | 2.45 (1.02–5.90) | 0.98(-0.21) |
| Romidepsin | 5 | 549 | 554 | 13.86 (5.73–33.49) | 2.13(0.95) |
| Lamivudine | 5 | 1,770 | 1,775 | 4.29 (1.78–10.35) | 1.47(0.28) |
| Docetaxel | 5 | 7,059 | 7,064 | 1.07 (0.45–2.58) | 0.08(-1.10) |
| Pneumococcal vaccine | 5 | 3,630 | 3,635 | 2.09 (0.87–5.04) | 0.82(-0.36) |
| Pazopanib hydrochloride | 5 | 2,452 | 2,457 | 3.1 (1.29–7.47) | 1.19(0.01) |
| Pregabalin | 5 | 7,988 | 7,993 | 0.95 (0.39–2.28) | -0.07(-1.25) |
| Bendamustine hydrochloride | 5 | 5,604 | 5,609 | 1.35 (0.56–3.26) | 0.35(-0.83) |
| Zanamivir hydrate | 5 | 1,279 | 1,284 | 5.94 (2.47–14.33) | 1.70(0.51) |
| Gefitinib | 5 | 3,047 | 3,052 | 2.49 (1.03–6.00) | 0.99(-0.19) |
| Sulfamethoxazole and trimethoprim | 5 | 4,918 | 4,923 | 1.54 (0.64–3.72) | 0.50(-0.68) |
| Axitinib | 5 | 2,363 | 2,368 | 3.22 (1.33–7.75) | 1.23(0.04) |
| Irinotecan hydrochloride | 5 | 16,017 | 16,022 | 0.47 (0.20–1.13) | -0.95(-2.13) |

ROR, reporting odds ratio; IC, information component; CI, confidence interval

Table S3. Shape parameter β of the Weibull distribution and failure pattern for each drug associated with taste disorders in the FAERS database.

| **Drug** | **Cases** | **Scale parameter α** | **Shape parameter β** | **Pattern** | **Min** | **Q1** | **Median** | **Q3** | **Max** | **Mean** |
| --- | --- | --- | --- | --- | --- | --- | --- | --- | --- | --- |
| NIRMATRELVIR/RITONAVIR | 3,968 | 1.69 | 0.88(0.85–0.91) | Early failure | 0.5 | 0.5 | 0.5 | 1 | 445 | 2.0 |
| SODIUM CITRATE | 1,129 | 1.71 | 3.65(0.05–7.24) | Random failure | 0.5 | 0.5 | 0.5 | 0.5 | 1 | 0.5 |
| LENALIDOMIDE | 1,072 | 142.81 | 0.52(0.51–0.53) | Early failure | 0.5 | 13 | 61 | 304 | 7,847 | 297.5 |
| CABOZANTINIB S-MALATE | 858 | 40.45 | 0.70(0.68–0.71) | Early failure | 0.5 | 9 | 23 | 53 | 1,741 | 59.4 |
| SUNITINIB MALATE | 514 | 41.92 | 0.53(0.51–0.55) | Early failure | 0.5 | 4 | 16.5 | 61 | 3,835 | 107.9 |
| VISMODEGIB | 481 | 59.26 | 0.61(0.59–0.63) | Early failure | 0.5 | 12 | 35 | 88 | 1,772 | 81.8 |
| CLARITHROMYCIN | 419 | 1.45 | 1.07(0.97–1.18) | Random failure | 0.5 | 0.5 | 0.5 | 1 | 45 | 1.4 |
| ENZALUTAMIDE | 358 | 83.81 | 0.56(0.54–0.58) | Early failure | 0.5 | 11.5 | 54.5 | 160 | 1,592 | 114.3 |
| ADALIMUMAB | 293 | 59.34 | 0.36(0.34–0.39) | Early failure | 0.5 | 0.5 | 13 | 166 | 3,686 | 238.1 |
| LIFITEGRAST | 261 | 4.17 | 0.49(0.42–0.55) | Early failure | 0.5 | 0.5 | 0.5 | 2 | 701 | 13.6 |
| PALBOCICLIB | 259 | 67.74 | 0.51(0.49–0.53) | Early failure | 0.5 | 6 | 29 | 150.5 | 2,066 | 131.3 |
| NINTEDANIB | 256 | 45.05 | 0.52(0.50–0.55) | Early failure | 0.5 | 3 | 21 | 81 | 1,145 | 90.1 |
| TERBINAFINE | 232 | 40.77 | 0.89(0.85–0.93) | Early failure | 0.5 | 17 | 32 | 42 | 229 | 35.1 |
| NIRAPARIB | 207 | 34.14 | 0.49(0.46–0.52) | Early failure | 0.5 | 0.5 | 14 | 80.5 | 743 | 68.6 |
| SECUKINUMAB | 202 | 134.29 | 0.40(0.38–0.43) | Early failure | 0.5 | 0.75 | 57 | 365 | 1,817 | 239.8 |
| FINGOLIMOD HYDROCHLORIDE | 200 | 19.45 | 0.31(0.27–0.36) | Early failure | 0.5 | 0.5 | 0.5 | 39 | 3,522 | 150.2 |
| PIRFENIDONE | 195 | 93.29 | 0.56(0.54–0.59) | Early failure | 0.5 | 13.5 | 41 | 128 | 3,242 | 157.6 |
| TERIPARATIDE | 184 | 18.82 | 0.46(0.42–0.50) | Early failure | 0.5 | 0.5 | 5 | 53 | 577 | 42.8 |
| OMEPRAZOLE | 174 | 7.20 | 0.38(0.32–0.45) | Early failure | 0.5 | 0.5 | 0.5 | 2 | 1,127 | 42.6 |
| EVEROLIMUS | 172 | 32.51 | 0.54(0.51–0.57) | Early failure | 0.5 | 1 | 14 | 58 | 851 | 67.2 |
| NIVOLUMAB | 165 | 50.01 | 0.57(0.54–0.60) | Early failure | 0.5 | 6 | 28 | 61 | 571 | 74.3 |
| RUCAPARIB CAMSYLATE | 162 | 13.15 | 0.59(0.55–0.64) | Early failure | 0.5 | 0.5 | 6 | 14 | 1,291 | 36.8 |
| AVAPRITINIB | 162 | 24.45 | 0.56(0.53–0.60) | Early failure | 0.5 | 1.5 | 13 | 35 | 703 | 48.4 |
| SEMAGLUTIDE | 156 | 18.84 | 0.47(0.43–0.51) | Early failure | 0.5 | 0.5 | 6.5 | 43 | 910 | 43.5 |
| PAZOPANIB HYDROCHLORIDE | 155 | 42.33 | 0.55(0.52–0.58) | Early failure | 0.5 | 2.5 | 18 | 85 | 1,202 | 81.3 |
| DUPILUMAB | 149 | 66.36 | 0.41(0.38–0.44) | Early failure | 0.5 | 0.5 | 39 | 139 | 1,386 | 157.7 |
| METRONIDAZOLE | 148 | 1.75 | 1.05(0.91–1.19) | Random failure | 0.5 | 0.5 | 1 | 2 | 27 | 1.8 |
| CAPECITABINE | 145 | 43.56 | 0.62(0.59–0.65) | Early failure | 0.5 | 6 | 31 | 70 | 481 | 52.8 |
| APREMILAST | 133 | 31.11 | 0.48(0.45–0.52) | Early failure | 0.5 | 0.5 | 9 | 61 | 1,429 | 80.6 |
| AZELASTINE HYDROCHLORIDE | 117 | 2.78 | 0.46(0.32–0.61) | Early failure | 0.5 | 0.5 | 0.5 | 0.5 | 366 | 10.8 |
| OCRELIZUMAB | 113 | 119.25 | 0.32(0.28–0.35) | Early failure | 0.5 | 0.5 | 76 | 407 | 2,026 | 316.3 |
| FLUTICASONE PROPIONATE | 110 | 6.80 | 0.48(0.40–0.56) | Early failure | 0.5 | 0.5 | 1 | 4 | 889 | 27.6 |
| POMALIDOMIDE | 105 | 168.17 | 0.48(0.46–0.51) | Early failure | 0.5 | 13 | 78 | 367 | 2,331 | 265.0 |
| TERBINAFINE HYDROCHLORIDE | 104 | 39.89 | 0.86(0.80–0.91) | Early failure | 0.5 | 18.5 | 30 | 46 | 259 | 36.0 |
| PACLITAXEL | 98 | 22.45 | 0.54(0.50–0.59) | Early failure | 0.5 | 0.5 | 14 | 39.5 | 188 | 32.0 |
| ETANERCEPT | 97 | 46.62 | 0.38(0.34–0.42) | Early failure | 0.5 | 0.5 | 12 | 108 | 5,717 | 276.4 |
| TREPROSTINIL | 95 | 129.02 | 0.50(0.47–0.52) | Early failure | 0.5 | 8 | 53 | 360.5 | 2,324 | 251.0 |
| PEMBROLIZUMAB | 91 | 35.01 | 0.54(0.50–0.58) | Early failure | 0.5 | 2 | 15 | 81 | 655 | 60.0 |
| CIPROFLOXACIN | 91 | 3.98 | 0.63(0.52–0.73) | Early failure | 0.5 | 0.5 | 1 | 7 | 118 | 7.0 |
| LENVATINIB | 91 | 23.89 | 0.75(0.71–0.79) | Early failure | 0.5 | 4 | 12 | 30 | 495 | 38.0 |
| DULAGLUTIDE | 89 | 10.09 | 0.45(0.37–0.52) | Early failure | 0.5 | 0.5 | 1 | 20 | 365 | 26.4 |
| SERTRALINE HYDROCHLORIDE | 86 | 31.42 | 0.38(0.33–0.43) | Early failure | 0.5 | 0.5 | 2.5 | 71 | 936 | 117.1 |
| SODIUM OXYBATE | 85 | 61.87 | 0.32(0.28–0.36) | Early failure | 0.5 | 0.5 | 7 | 77 | 4,644 | 482.2 |
| RIBOCICLIB | 82 | 66.99 | 0.44(0.40–0.48) | Early failure | 0.5 | 1 | 25.5 | 145 | 1,917 | 147.6 |
| APOMORPHINE HYDROCHLORIDE | 80 | 3.94 | 0.35(0.19–0.51) | Early failure | 0.5 | 0.5 | 0.5 | 0.5 | 1,053 | 26.5 |
| NICOTINE | 77 | 2.72 | 0.55(0.39–0.71) | Early failure | 0.5 | 0.5 | 0.5 | 0.5 | 76 | 4.6 |
| CRIZOTINIB | 76 | 36.60 | 0.50(0.46–0.55) | Early failure | 0.5 | 2 | 9.5 | 59 | 1,492 | 111.6 |
| LEVOFLOXACIN | 75 | 2.90 | 0.79(0.66–0.92) | Early failure | 0.5 | 0.5 | 1 | 3.5 | 18 | 3.3 |
| TOFACITINIB CITRATE | 71 | 92.09 | 0.44(0.41–0.48) | Early failure | 0.5 | 5.5 | 51 | 153 | 1,600 | 180.6 |
| DIMETHYL FUMARATE | 70 | 69.28 | 0.42(0.38–0.46) | Early failure | 0.5 | 3 | 13 | 153 | 2,603 | 246.4 |
| OMALIZUMAB | 69 | 49.99 | 0.34(0.29–0.39) | Early failure | 0.5 | 0.5 | 14 | 87 | 2,246 | 226.4 |
| TOPIRAMATE | 68 | 10.45 | 0.52(0.44–0.60) | Early failure | 0.5 | 0.5 | 4 | 17 | 644 | 35.5 |
| OXALIPLATIN | 67 | 13.31 | 0.68(0.62–0.75) | Early failure | 0.5 | 1 | 11 | 19 | 133 | 15.3 |
| EVOLOCUMAB | 66 | 15.83 | 0.42(0.35–0.49) | Early failure | 0.5 | 0.5 | 2 | 25 | 341 | 41.7 |
| OFATUMUMAB | 65 | 16.08 | 0.36(0.29–0.44) | Early failure | 0.5 | 0.5 | 0.5 | 43 | 568 | 53.1 |
| ALBUTEROL SULFATE | 64 | 2.29 | 0.47(0.22–0.71) | Early failure | 0.5 | 0.5 | 0.5 | 0.5 | 730 | 12.4 |
| CARBOPLATIN | 64 | 26.65 | 0.46(0.40–0.52) | Early failure | 0.5 | 0.5 | 11 | 53 | 3,001 | 125.5 |
| PEGINTERFERON ALFA-2A | 64 | 18.94 | 0.51(0.45–0.57) | Early failure | 0.5 | 0.5 | 6.5 | 43 | 268 | 36.0 |
| ERLOTINIB HYDROCHLORIDE | 63 | 70.11 | 0.60(0.56–0.64) | Early failure | 0.5 | 14.5 | 39 | 80 | 621 | 89.8 |
| GLATIRAMER ACETATE | 63 | 234.43 | 0.35(0.31–0.38) | Early failure | 0.5 | 0.75 | 112 | 639 | 7,502 | 637.5 |
| POLYETHYLENE GLYCOL 3,350 | 61 | 5.15 | 0.36(0.23–0.50) | Early failure | 0.5 | 0.5 | 0.5 | 0.5 | 610 | 25.4 |
| TERIFLUNOMIDE | 61 | 101.31 | 0.55(0.52–0.57) | Early failure | 0.5 | 9 | 46 | 197 | 1,195 | 183.3 |
| ENFORTUMAB VEDOTIN-EJFV | 61 | 23.78 | 0.77(0.74–0.81) | Early failure | 0.5 | 6 | 13 | 35 | 196 | 28.0 |
| SACUBITRIL\VALSARTAN | 61 | 52.06 | 0.42(0.37–0.48) | Early failure | 0.5 | 0.5 | 31 | 92 | 718 | 86.9 |
| LIRAGLUTIDE | 59 | 9.39 | 0.46(0.37–0.54) | Early failure | 0.5 | 0.5 | 1 | 12.5 | 433 | 29.6 |
| DOCETAXEL | 58 | 15.00 | 0.72(0.67–0.78) | Early failure | 0.5 | 2 | 7 | 22 | 99 | 17.4 |
| FLUOROURACIL | 57 | 17.69 | 0.71(0.66–0.76) | Early failure | 0.5 | 3 | 11 | 30 | 189 | 23.4 |
| IBRUTINIB | 56 | 101.66 | 0.37(0.32–0.41) | Early failure | 0.5 | 0.75 | 24.5 | 422.5 | 2,710 | 313.8 |
| VEDOLIZUMAB | 56 | 151.99 | 0.42(0.38–0.46) | Early failure | 0.5 | 11 | 81.5 | 437.5 | 1,641 | 255.2 |
| CERTOLIZUMAB PEGOL | 55 | 54.60 | 0.36(0.31–0.41) | Early failure | 0.5 | 0.5 | 9 | 185 | 2,984 | 238.0 |
| IBUPROFEN | 55 | 1.74 | 0.58(0.28–0.89) | Early failure | 0.5 | 0.5 | 0.5 | 0.5 | 77 | 2.6 |
| AMLODIPINE BESYLATE | 55 | 23.93 | 0.40(0.33–0.47) | Early failure | 0.5 | 0.5 | 2 | 23 | 1,719 | 113.6 |
| ABEMACICLIB | 55 | 27.07 | 0.57(0.52–0.62) | Early failure | 0.5 | 4 | 16 | 54 | 365 | 41.2 |
| ABALOPARATIDE | 54 | 10.39 | 0.44(0.35–0.53) | Early failure | 0.5 | 0.5 | 0.5 | 17.5 | 287 | 25.3 |
| AFATINIB | 54 | 22.81 | 0.55(0.49–0.61) | Early failure | 0.5 | 2.5 | 7.5 | 28 | 891 | 59.6 |
| DENOSUMAB | 53 | 38.47 | 0.40(0.35–0.46) | Early failure | 0.5 | 0.5 | 8 | 113 | 1,102 | 112.6 |
| LEUPROLIDE ACETATE | 53 | 80.21 | 0.39(0.34–0.43) | Early failure | 0.5 | 0.5 | 33 | 161 | 2,022 | 217.0 |
| ZOLEDRONIC ACID | 53 | 20.72 | 0.32(0.24–0.39) | Early failure | 0.5 | 0.5 | 0.5 | 29 | 2,947 | 192.3 |
| MACITENTAN | 53 | 212.72 | 0.50(0.47–0.53) | Early failure | 0.5 | 22 | 108 | 588 | 1,666 | 330.7 |
| CARBIDOPA\LEVODOPA | 52 | 474.45 | 0.37(0.34–0.41) | Early failure | 0.5 | 12.5 | 339 | 1,210 | 3,468 | 734.7 |
| VARENICLINE TARTRATE | 52 | 13.41 | 0.52(0.45–0.60) | Early failure | 0.5 | 0.5 | 6 | 17 | 1,248 | 43.7 |
| TRASTUZUMAB | 50 | 32.37 | 0.44(0.38–0.49) | Early failure | 0.5 | 0.75 | 7 | 41 | 2,155 | 138.6 |
| AZELASTINE HYDROCHLORIDE\FLUTICASONE PROPIONATE | 47 | 3.21 | 0.48(0.30–0.67) | Early failure | 0.5 | 0.5 | 0.5 | 0.5 | 104 | 6.3 |
| OCTREOTIDE ACETATE | 44 | 414.79 | 0.36(0.33–0.40) | Early failure | 0.5 | 21 | 103 | 1,495 | 8,140 | 1,059.5 |
| DICHLORPHENAMIDE | 44 | 15.49 | 0.50(0.42–0.58) | Early failure | 0.5 | 0.75 | 4 | 16 | 1,678 | 72.5 |
| BEVACIZUMAB | 44 | 55.83 | 0.45(0.41–0.50) | Early failure | 0.5 | 2.5 | 24 | 124 | 1,243 | 119.0 |
| RIVAROXABAN | 44 | 12.66 | 0.52(0.44–0.61) | Early failure | 0.5 | 0.5 | 3.5 | 19.5 | 145 | 21.5 |
| CHLORHEXIDINE GLUCONATE | 44 | 3.79 | 0.48(0.31–0.65) | Early failure | 0.5 | 0.5 | 0.5 | 2 | 194 | 11.3 |
| MARIBAVIR | 43 | 33.12 | 0.40(0.33–0.47) | Early failure | 0.5 | 0.5 | 4 | 106.5 | 489 | 77.9 |
| IMATINIB MESYLATE | 43 | 134.29 | 0.40(0.36–0.43) | Early failure | 0.5 | 3.5 | 77 | 381 | 3,653 | 366.5 |
| TIRZEPATIDE | 42 | 42.25 | 0.39(0.32–0.46) | Early failure | 0.5 | 0.5 | 16.5 | 142.5 | 294 | 73.2 |
| NALTREXONE | 42 | 4.90 | 0.51(0.37–0.65) | Early failure | 0.5 | 0.5 | 0.5 | 8 | 148 | 10.4 |
| CETYLPYRIDINIUM CHLORIDE | 42 | 1.74 | 0.94(0.67–1.20) | Random failure | 0.5 | 0.5 | 0.75 | 1 | 37 | 2.1 |
| FLUTICASONE FUROATE\VILANTEROL TRIFENATATE | 42 | 25.02 | 0.41(0.34–0.48) | Early failure | 0.5 | 0.75 | 6 | 37.5 | 5,585 | 224.8 |
| ALPELISIB | 40 | 32.06 | 0.62(0.57–0.68) | Early failure | 0.5 | 7 | 17.5 | 47 | 180 | 35.4 |
| LETROZOLE | 40 | 56.09 | 0.37(0.31–0.43) | Early failure | 0.5 | 0.5 | 11.5 | 53.5 | 3,642 | 249.8 |
| UMECLIDINIUM BROMIDE\VILANTEROL TRIFENATATE | 40 | 7.22 | 0.42(0.29–0.54) | Early failure | 0.5 | 0.5 | 0.5 | 10.5 | 222 | 21.4 |
| ESKETAMINE | 40 | 8.77 | 0.35(0.23–0.47) | Early failure | 0.5 | 0.5 | 0.5 | 5.5 | 1,204 | 55.4 |
| APIXABAN | 39 | 37.95 | 0.44(0.38–0.50) | Early failure | 0.5 | 0.75 | 17 | 72 | 1,095 | 96.3 |
| OSELTAMIVIR PHOSPHATE | 39 | 2.11 | 0.94(0.72–1.16) | Random failure | 0.5 | 0.5 | 1 | 2 | 22 | 2.3 |
| ENFORTUMAB VEDOTIN | 39 | 21.25 | 0.89(0.81–0.97) | Early failure | 0.5 | 7 | 14 | 20.5 | 127 | 22.5 |
| DULOXETINE HYDROCHLORIDE | 39 | 21.78 | 0.37(0.29–0.45) | Early failure | 0.5 | 0.5 | 2 | 27.5 | 3,282 | 142.1 |
| SORAFENIB | 38 | 24.34 | 0.52(0.45–0.58) | Early failure | 0.5 | 2 | 10 | 29 | 1,081 | 75.3 |
| FLUTICASONE PROPIONATE\SALMETEROL XINAFOATE | 38 | 28.36 | 0.29(0.21–0.37) | Early failure | 0.5 | 0.5 | 0.5 | 18.5 | 5,097 | 351.9 |
| CYCLOSPORINE | 38 | 26.44 | 0.40(0.33–0.47) | Early failure | 0.5 | 0.5 | 8.5 | 60 | 2,741 | 113.8 |
| MAGNESIUM SULFATE\POTASSIUM SULFATE\SODIUM SULFATE | 38 | 1.00 | 1.02(0.35–1.68) | Random failure | 0.5 | 0.5 | 0.5 | 0.5 | 8 | 0.8 |
| ENTRECTINIB | 38 | 10.42 | 0.67(0.55–0.78) | Early failure | 0.5 | 3 | 4.5 | 7.5 | 740 | 30.7 |
| REGORAFENIB | 37 | 7.54 | 0.49(0.37–0.61) | Early failure | 0.5 | 0.5 | 1 | 9 | 460 | 24.6 |
| BUDESONIDE | 37 | 32.12 | 0.49(0.43–0.55) | Early failure | 0.5 | 3 | 14 | 40 | 838 | 77.2 |
| APALUTAMIDE | 37 | 40.84 | 0.62(0.58–0.67) | Early failure | 0.5 | 7 | 23 | 52 | 665 | 56.7 |
| AXITINIB | 36 | 111.83 | 0.53(0.50–0.56) | Early failure | 0.5 | 10 | 62.5 | 227 | 709 | 155.2 |
| VEMURAFENIB | 36 | 15.35 | 0.67(0.60–0.74) | Early failure | 0.5 | 4 | 7.5 | 25 | 159 | 19.4 |
| ERENUMAB-AOOE | 36 | 6.76 | 0.45(0.32–0.58) | Early failure | 0.5 | 0.5 | 0.5 | 11.5 | 109 | 14.7 |
| LEVOTHYROXINE SODIUM | 36 | 21.07 | 0.42(0.34–0.50) | Early failure | 0.5 | 0.5 | 5 | 40.5 | 716 | 49.0 |
| AZITHROMYCIN ANHYDROUS | 35 | 8.65 | 0.57(0.46–0.68) | Early failure | 0.5 | 0.5 | 3 | 20 | 46 | 11.9 |
| OXYMETAZOLINE HYDROCHLORIDE | 35 | 2.59 | 0.61(0.38–0.85) | Early failure | 0.5 | 0.5 | 0.5 | 1 | 93 | 5.5 |
| ONABOTULINUMTOXINA | 35 | 12.53 | 0.36(0.26–0.47) | Early failure | 0.5 | 0.5 | 1 | 7.5 | 1,484 | 107.9 |
| FLUTICASONE FUROATE\UMECLIDINIUM BROMIDE\VILANTEROL TRIFENATATE | 34 | 35.57 | 0.35(0.28–0.42) | Early failure | 0.5 | 0.5 | 4 | 68.5 | 2,039 | 157.9 |
| PANITUMUMAB | 33 | 76.20 | 0.60(0.57–0.63) | Early failure | 0.5 | 12 | 49 | 112 | 406 | 88.8 |
| TIPIRACIL HYDROCHLORIDE\TRIFLURIDINE | 33 | 56.86 | 0.74(0.71–0.78) | Early failure | 0.5 | 9 | 34 | 100 | 201 | 58.8 |
| DICLOFENAC SODIUM | 33 | 2.52 | 0.53(0.27–0.79) | Early failure | 0.5 | 0.5 | 0.5 | 0.5 | 37 | 3.3 |
| FLUTICASONE FUROATE | 32 | 6.97 | 0.52(0.39–0.64) | Early failure | 0.5 | 0.5 | 1 | 8 | 270 | 16.8 |
| NATALIZUMAB | 32 | 550.23 | 0.29(0.25–0.34) | Early failure | 0.5 | 1 | 316.5 | 1,753.5 | 4,950 | 1,013.1 |
| DASABUVIR\OMBITASVIR\PARITAPREVIR\RITONAVIR | 32 | 22.37 | 0.65(0.60–0.71) | Early failure | 0.5 | 2.5 | 10 | 40 | 366 | 32.7 |
| PHENTERMINE HYDROCHLORIDE\TOPIRAMATE | 32 | 8.83 | 0.45(0.33–0.57) | Early failure | 0.5 | 0.5 | 0.5 | 16.5 | 152 | 19.4 |
| METFORMIN HYDROCHLORIDE | 31 | 181.27 | 0.30(0.25–0.36) | Early failure | 0.5 | 0.5 | 31 | 788 | 2,496 | 591.8 |
| LEVONORGESTREL | 31 | 8.88 | 0.38(0.25–0.51) | Early failure | 0.5 | 0.5 | 0.5 | 4.5 | 595 | 48.5 |
| LINEZOLID | 31 | 11.02 | 0.70(0.60–0.80) | Early failure | 0.5 | 2 | 3 | 29 | 47 | 13.2 |
| ATORVASTATIN | 30 | 72.39 | 0.39(0.33–0.45) | Early failure | 0.5 | 2 | 30 | 135 | 4,523 | 325.7 |
| TIOTROPIUM BROMIDE MONOHYDRATE | 30 | 16.08 | 0.45(0.35–0.54) | Early failure | 0.5 | 0.5 | 5 | 30.5 | 214 | 29.1 |
| RITUXIMAB | 30 | 40.53 | 0.47(0.42–0.53) | Early failure | 0.5 | 3 | 13.5 | 68 | 605 | 80.4 |
| DABRAFENIB MESYLATE | 30 | 92.51 | 0.55(0.51–0.60) | Early failure | 0.5 | 10.5 | 32 | 103 | 1,571 | 173.5 |
| ABIRATERONE ACETATE | 30 | 57.66 | 0.55(0.49–0.62) | Early failure | 0.5 | 10 | 42.5 | 66 | 730 | 68.9 |
| CLOPIDOGREL BISULFATE | 30 | 91.91 | 0.48(0.41–0.55) | Early failure | 0.5 | 31 | 56 | 153 | 3,471 | 182.6 |
| CORTICOTROPIN | 30 | 49.40 | 0.40(0.34–0.46) | Early failure | 0.5 | 1 | 10 | 106.5 | 2,892 | 183.6 |
| OSIMERTINIB | 29 | 53.30 | 0.83(0.77–0.9) | Early failure | 1 | 10 | 24 | 76 | 552 | 66.9 |
| ABATACEPT | 29 | 226.84 | 0.33(0.28–0.39) | Early failure | 0.5 | 1 | 64 | 1,009 | 4,222 | 657.6 |
| HUMAN IMMUNOGLOBULIN G | 29 | 301.77 | 0.31(0.25–0.36) | Early failure | 0.5 | 0.5 | 167 | 880 | 7,028 | 822.3 |
| AMOXICILLIN | 28 | 3.74 | 0.75(0.56–0.94) | Early failure | 0.5 | 0.5 | 1.5 | 5 | 37 | 4.8 |
| ATEZOLIZUMAB | 28 | 21.60 | 0.55(0.47–0.64) | Early failure | 0.5 | 1 | 8 | 34.5 | 361 | 39.9 |
| IXAZOMIB | 28 | 41.82 | 0.60(0.54–0.67) | Early failure | 0.5 | 4.5 | 13.5 | 86 | 437 | 69.5 |
| PRALSETINIB | 28 | 25.52 | 0.71(0.63–0.79) | Early failure | 0.5 | 5 | 14.5 | 25 | 260 | 34.5 |
| AMIKACIN | 28 | 6.54 | 0.41(0.24–0.58) | Early failure | 0.5 | 0.5 | 0.5 | 4 | 977 | 44.4 |
| AMBRISENTAN | 28 | 658.65 | 0.51(0.46–0.56) | Early failure | 0.5 | 84 | 371 | 1,395.5 | 3,081 | 783.1 |
| TEPROTUMUMAB-TRBW | 27 | 44.70 | 0.50(0.42–0.58) | Early failure | 0.5 | 2 | 43 | 77 | 430 | 59.6 |
| CITALOPRAM HYDROBROMIDE | 27 | 104.08 | 0.31(0.23–0.38) | Early failure | 0.5 | 0.5 | 12 | 618 | 1,095 | 293.0 |
| LEDIPASVIR\SOFOSBUVIR | 27 | 37.80 | 0.68(0.59–0.77) | Early failure | 0.5 | 16 | 27 | 53.5 | 181 | 36.6 |
| CLINDAMYCIN | 27 | 2.17 | 0.75(0.45–1.05) | Random failure | 0.5 | 0.5 | 0.5 | 2.5 | 28 | 2.6 |
| ZINC ACETATE ANHYDROUS\ZINC GLUCONATE | 27 | 3.22 | 0.88(0.67–1.10) | Random failure | 0.5 | 0.75 | 1 | 4 | 51 | 4.2 |
| ACETAMINOPHEN | 27 | 1.66 | 0.67(0.24–1.10) | Random failure | 0.5 | 0.5 | 0.5 | 0.5 | 19 | 1.7 |
| FENTANYL | 26 | 8.15 | 0.41(0.25–0.57) | Early failure | 0.5 | 0.5 | 1 | 3 | 730 | 42.9 |
| OLAPARIB | 26 | 24.62 | 0.54(0.45–0.63) | Early failure | 0.5 | 2 | 8.5 | 15 | 1,052 | 71.9 |
| ALEMTUZUMAB | 26 | 5.85 | 0.47(0.30–0.65) | Early failure | 0.5 | 0.5 | 0.75 | 4 | 608 | 29.7 |
| INSULIN GLARGINE | 26 | 50.35 | 0.37(0.29–0.45) | Early failure | 0.5 | 0.5 | 13 | 61.5 | 3,472 | 288.6 |
| IRINOTECAN | 26 | 27.87 | 0.58(0.49–0.66) | Early failure | 0.5 | 5.5 | 16.5 | 53.5 | 100 | 32.2 |
| VENETOCLAX | 26 | 51.15 | 0.46(0.39–0.54) | Early failure | 0.5 | 4.5 | 11.5 | 117 | 880 | 112.2 |
| NILOTINIB | 26 | 449.05 | 0.56(0.52–0.61) | Early failure | 0.5 | 67.5 | 315.5 | 587 | 3,434 | 515.6 |
| IXAZOMIB CITRATE | 25 | 101.47 | 0.71(0.62–0.80) | Early failure | 0.5 | 23 | 48 | 113 | 1,294 | 146.0 |
| SODIUM FLUORIDE | 25 | 11.82 | 0.49(0.36–0.61) | Early failure | 0.5 | 0.5 | 4 | 16 | 365 | 34.8 |
| INTERFERON BETA-1A | 25 | 608.97 | 0.34(0.30–0.39) | Early failure | 0.5 | 7 | 190 | 2,292 | 7,037 | 1,420.2 |
| RISANKIZUMAB-RZAA | 25 | 132.47 | 0.44(0.37–0.51) | Early failure | 0.5 | 28 | 101 | 214 | 821 | 159.4 |
| CABOZANTINIB | 25 | 21.49 | 0.89(0.81–0.97) | Early failure | 0.5 | 6 | 17 | 28 | 62 | 19.6 |
| MOMETASONE FUROATE | 25 | 36.30 | 0.49(0.41–0.57) | Early failure | 0.5 | 1 | 11 | 81 | 730 | 93.3 |
| DASATINIB | 24 | 109.69 | 0.43(0.37–0.49) | Early failure | 0.5 | 5 | 56 | 177 | 1,864 | 230.0 |
| DEXAMETHASONE | 24 | 28.03 | 0.45(0.36–0.55) | Early failure | 0.5 | 1 | 7 | 45.5 | 487 | 66.4 |
| VENLAFAXINE HYDROCHLORIDE | 24 | 14.51 | 0.40(0.28–0.53) | Early failure | 0.5 | 0.5 | 0.75 | 12.5 | 351 | 47.5 |
| RIBAVIRIN | 24 | 6.47 | 0.53(0.37–0.69) | Early failure | 0.5 | 0.5 | 1.25 | 12 | 65 | 11.3 |
| AMPHETAMINE ASPARTATE\AMPHETAMINE SULFATE\DEXTROAMPHETAMINE SACCHARATE\DEXTROAMPHETAMINE SULFATE | 24 | 10.90 | 0.36(0.21–0.50) | Early failure | 0.5 | 0.5 | 0.5 | 6.5 | 1,262 | 81.6 |
| ALIROCUMAB | 24 | 15.56 | 0.47(0.35–0.58) | Early failure | 0.5 | 0.5 | 5.5 | 35.5 | 505 | 37.8 |
| BAMLANIVIMAB | 24 | 2.99 | 0.64(0.39–0.89) | Early failure | 0.5 | 0.5 | 0.5 | 2.5 | 49 | 4.7 |
| CETUXIMAB | 24 | 17.00 | 0.54(0.45–0.64) | Early failure | 0.5 | 0.75 | 6.5 | 25 | 238 | 33.1 |
| ALECTINIB HYDROCHLORIDE | 23 | 28.18 | 0.62(0.55–0.69) | Early failure | 0.5 | 3 | 13 | 34 | 486 | 46.9 |
| SOFOSBUVIR\VELPATASVIR | 23 | 14.97 | 0.51(0.39–0.63) | Early failure | 0.5 | 0.5 | 11 | 25 | 100 | 19.8 |
| ESZOPICLONE | 23 | 1.19 | 1.11(0.53–1.68) | Random failure | 0.5 | 0.5 | 0.5 | 1 | 7 | 1.0 |
| TOCILIZUMAB | 23 | 87.79 | 0.40(0.33–0.47) | Early failure | 0.5 | 1.25 | 29 | 101 | 2,236 | 234.5 |
| DEXTROMETHORPHAN HYDROBROMIDE\GUAIFENESIN | 22 | 2.44 | 0.66(0.36–0.97) | Early failure | 0.5 | 0.5 | 0.5 | 1.5 | 30 | 3.2 |
| UPADACITINIB | 22 | 121.51 | 0.60(0.54–0.65) | Early failure | 0.5 | 14.5 | 96.5 | 129.5 | 1,691 | 183.6 |
| GADOTERATE MEGLUMINE | 22 | 1.57 | 0.64(0.09–1.19) | Random failure | 0.5 | 0.5 | 0.5 | 0.5 | 22 | 1.6 |
| FERRIC CARBOXYMALTOSE | 22 | 2.18 | 0.73(0.40–1.06) | Random failure | 0.5 | 0.5 | 0.5 | 1.5 | 24 | 2.7 |
| HOMEOPATHICS\ZINC ACETATE\ZINC GLUCONATE | 22 | 6.30 | 0.44(0.25–0.63) | Early failure | 0.5 | 0.5 | 0.75 | 2.5 | 365 | 31.6 |
| CETIRIZINE HYDROCHLORIDE | 22 | 2.97 | 0.45(0.13–0.77) | Early failure | 0.5 | 0.5 | 0.5 | 0.5 | 184 | 9.6 |
| CLINDAMYCIN HYDROCHLORIDE | 22 | 2.48 | 0.89(0.62–1.16) | Random failure | 0.5 | 0.5 | 1 | 3 | 11 | 2.6 |
| VORTIOXETINE HYDROBROMIDE | 22 | 32.78 | 0.49(0.41–0.57) | Early failure | 0.5 | 1.5 | 11.5 | 66 | 386 | 61.6 |
| LORATADINE | 22 | 6.80 | 0.36(0.16–0.56) | Early failure | 0.5 | 0.5 | 0.5 | 2 | 780 | 54.7 |
| LISINOPRIL | 21 | 221.41 | 0.40(0.34–0.46) | Early failure | 0.5 | 11 | 315 | 315 | 3,671 | 404.5 |
| EMPAGLIFLOZIN | 21 | 20.09 | 0.56(0.46–0.66) | Early failure | 0.5 | 2 | 5 | 45 | 273 | 37.4 |
| SELEXIPAG | 21 | 21.09 | 0.41(0.29–0.52) | Early failure | 0.5 | 0.5 | 1 | 53 | 399 | 56.6 |
| PREGABALIN | 21 | 40.69 | 0.34(0.24–0.43) | Early failure | 0.5 | 0.5 | 5 | 66 | 2,821 | 283.8 |
| ESOMEPRAZOLE MAGNESIUM | 21 | 7.79 | 0.38(0.19–0.56) | Early failure | 0.5 | 0.5 | 0.5 | 6 | 214 | 26.3 |
| GOLIMUMAB | 21 | 327.56 | 0.48(0.45–0.51) | Early failure | 0.5 | 61 | 126 | 512 | 2,861 | 545.1 |
| GABAPENTIN | 21 | 5.33 | 0.46(0.25–0.67) | Early failure | 0.5 | 0.5 | 0.5 | 3 | 138 | 13.8 |
| CIPROFLOXACIN HYDROCHLORIDE | 21 | 4.55 | 0.66(0.45–0.86) | Early failure | 0.5 | 0.5 | 2 | 4 | 151 | 10.1 |
| NAPROXEN SODIUM | 21 | 3.70 | 0.39(0.09–0.68) | Early failure | 0.5 | 0.5 | 0.5 | 0.5 | 152 | 10.2 |
| MIRTAZAPINE | 21 | 12.03 | 0.39(0.23–0.55) | Early failure | 0.5 | 0.5 | 3 | 9 | 7,488 | 361.4 |
| LANSOPRAZOLE | 20 | 124.40 | 0.34(0.26–0.42) | Early failure | 0.5 | 1.5 | 11.5 | 652 | 652 | 277.4 |
| RIOCIGUAT | 20 | 123.26 | 0.70(0.60–0.80) | Early failure | 2 | 13 | 101 | 224.5 | 339 | 129.7 |
| DALFAMPRIDINE | 20 | 270.43 | 0.45(0.41–0.49) | Early failure | 0.5 | 12 | 126 | 771 | 1,948 | 430.4 |
| ZINC ACETATE\ZINC GLUCONATE | 20 | 1.17 | 1.01(0.33–1.68) | Random failure | 0.5 | 0.5 | 0.5 | 0.75 | 8 | 1.0 |
| PREDNISOLONE | 20 | 9.37 | 0.59(0.44–0.74) | Early failure | 0.5 | 0.75 | 5 | 8 | 92 | 16.9 |

Table S4. ROR of taste disorders adjusted for age and sex using the FAERS database.

| **Drug** | **Cases** | **Total** | **Crude ROR (95%CI)** | **Adjusted ROR (95%CI)** | **Age** | **Sex** |
| --- | --- | --- | --- | --- | --- | --- |
| NIRMATRELVIR/RITONAVIR | 3,968 | 76,075 | 27.04 (26.11–27.99) | 26.02 (25.12–26.94) | 1.01 (1.01–1.01) | 1.13 (1.10–1.16) |
| SODIUM CITRATE | 1,129 | 30,742 | 16.52 (15.55–17.56) | 22.98 (21.57–24.48) | 1.01 (1.01–1.02) | 1.16 (1.13–1.19) |
| LENALIDOMIDE | 1,072 | 225,001 | 2.03 (1.91–2.16) | 1.78 (1.67–1.89) | 1.01 (1.01–1.01) | 1.17 (1.14–1.20) |
| CABOZANTINIB S-MALATE | 858 | 41,166 | 9.11 (8.50–9.76) | 8.87 (8.27–9.51) | 1.01 (1.01–1.01) | 1.21 (1.18–1.25) |
| SUNITINIB MALATE | 514 | 29,099 | 7.59 (6.95–8.29) | 7.25 (6.64–7.92) | 1.01 (1.01–1.01) | 1.19 (1.16–1.22) |
| VISMODEGIB | 481 | 5,801 | 38.22 (34.79–42.00) | 34.46 (31.35–37.89) | 1.01 (1.01–1.01) | 1.18 (1.15–1.21) |
| CLARITHROMYCIN | 419 | 14,906 | 12.18 (11.05–13.44) | 12.66 (11.47–13.96) | 1.01 (1.01–1.01) | 1.16 (1.13–1.19) |
| ENZALUTAMIDE | 358 | 52,107 | 2.90 (2.61–3.22) | 2.62 (2.36–2.92) | 1.01 (1.01–1.01) | 1.19 (1.16–1.23) |
| ADALIMUMAB | 293 | 365,776 | 0.32 (0.29–0.36) | 0.34 (0.30–0.38) | 1.01 (1.01–1.01) | 1.17 (1.14–1.20) |
| LIFITEGRAST | 261 | 5,191 | 22.18 (19.57–25.13) | 19.32 (17.04–21.90) | 1.01 (1.01–1.01) | 1.15 (1.12–1.18) |
| PALBOCICLIB | 259 | 67,780 | 1.60 (1.41–1.81) | 1.37 (1.21–1.55) | 1.01 (1.01–1.01) | 1.16 (1.13–1.19) |
| NINTEDANIB | 256 | 46,468 | 2.31 (2.04–2.61) | 2.01 (1.77–2.27) | 1.01 (1.01–1.01) | 1.17 (1.14–1.20) |
| TERBINAFINE | 232 | 3,618 | 28.67 (25.09–32.77) | 28.96 (25.33–33.10) | 1.01 (1.01–1.01) | 1.17 (1.14–1.20) |
| NIRAPARIB | 207 | 36,015 | 2.41 (2.10–2.76) | 2.06 (1.80–2.37) | 1.01 (1.01–1.01) | 1.16 (1.13–1.19) |
| SECUKINUMAB | 202 | 118,433 | 0.71 (0.61–0.81) | 0.73 (0.64–0.84) | 1.01 (1.01–1.01) | 1.17 (1.14–1.20) |
| FINGOLIMOD HYDROCHLORIDE | 200 | 99,965 | 0.83 (0.72–0.95) | 0.93 (0.81–1.07) | 1.01 (1.01–1.01) | 1.17 (1.14–1.20) |
| PIRFENIDONE | 195 | 27,643 | 2.96 (2.57–3.41) | 2.56 (2.22–2.95) | 1.01 (1.01–1.01) | 1.17 (1.14–1.20) |
| TERIPARATIDE | 184 | 69,940 | 1.09 (0.95–1.27) | 0.90 (0.78–1.04) | 1.01 (1.01–1.01) | 1.17 (1.14–1.20) |
| OMEPRAZOLE | 174 | 14,937 | 4.92 (4.23–5.71) | 4.86 (4.18–5.65) | 1.01 (1.01–1.01) | 1.17 (1.14–1.20) |
| EVEROLIMUS | 172 | 38,401 | 1.87 (1.61–2.18) | 1.87 (1.61–2.17) | 1.01 (1.01–1.01) | 1.17 (1.14–1.20) |
| NIVOLUMAB | 165 | 87,607 | 0.78 (0.67–0.91) | 0.74 (0.64–0.87) | 1.01 (1.01–1.01) | 1.16 (1.13–1.19) |
| AVAPRITINIB | 162 | 16,263 | 4.19 (3.59–4.90) | 3.84 (3.29–4.49) | 1.01 (1.01–1.01) | 1.17 (1.14–1.20) |
| RUCAPARIB CAMSYLATE | 162 | 7,689 | 8.98 (7.68–10.49) | 7.79 (6.66–9.11) | 1.01 (1.01–1.01) | 1.16 (1.13–1.19) |
| SEMAGLUTIDE | 156 | 30,362 | 2.15 (1.84–2.52) | 2.06 (1.76–2.41) | 1.01 (1.01–1.01) | 1.17 (1.14–1.20) |
| PAZOPANIB HYDROCHLORIDE | 155 | 18,559 | 3.51 (2.99–4.11) | 3.39 (2.89–3.97) | 1.01 (1.01–1.01) | 1.17 (1.14–1.20) |
| DUPILUMAB | 149 | 159,334 | 0.38 (0.33–0.45) | 0.42 (0.36–0.50) | 1.01 (1.01–1.01) | 1.17 (1.14–1.20) |
| METRONIDAZOLE | 148 | 9,818 | 6.38 (5.42–7.51) | 6.57 (5.58–7.73) | 1.01 (1.01–1.01) | 1.16 (1.13–1.19) |
| CAPECITABINE | 145 | 36,663 | 1.65 (1.40–1.94) | 1.52 (1.29–1.79) | 1.01 (1.01–1.01) | 1.17 (1.14–1.20) |
| APREMILAST | 133 | 85,328 | 0.65 (0.54–0.77) | 0.65 (0.55–0.77) | 1.01 (1.01–1.01) | 1.17 (1.14–1.20) |
| AZELASTINE HYDROCHLORIDE | 117 | 1,776 | 29.38 (24.35–35.45) | 27.33 (22.64–32.99) | 1.01 (1.01–1.01) | 1.16 (1.13–1.19) |
| OCRELIZUMAB | 113 | 82,225 | 0.57 (0.47–0.68) | 0.61 (0.51–0.74) | 1.01 (1.01–1.01) | 1.17 (1.14–1.20) |
| FLUTICASONE PROPIONATE | 110 | 7,703 | 6.03 (4.99–7.28) | 5.98 (4.95–7.22) | 1.01 (1.01–1.01) | 1.17 (1.13–1.20) |
| POMALIDOMIDE | 105 | 54,720 | 0.80 (0.66–0.96) | 0.70 (0.57–0.84) | 1.01 (1.01–1.01) | 1.17 (1.14–1.20) |
| TERBINAFINE HYDROCHLORIDE | 104 | 1,749 | 26.32 (21.58–32.10) | 26.28 (21.54–32.06) | 1.01 (1.01–1.01) | 1.17 (1.14–1.20) |
| PACLITAXEL | 98 | 38,050 | 1.07 (0.88–1.31) | 0.99 (0.82–1.21) | 1.01 (1.01–1.01) | 1.17 (1.14–1.20) |
| ETANERCEPT | 97 | 145,237 | 0.27 (0.22–0.33) | 0.27 (0.22–0.33) | 1.01 (1.01–1.01) | 1.17 (1.14–1.21) |
| TREPROSTINIL | 95 | 91,979 | 0.43 (0.35–0.52) | 0.41 (0.33–0.50) | 1.01 (1.01–1.01) | 1.17 (1.14–1.20) |
| CIPROFLOXACIN | 91 | 41,033 | 0.92 (0.75–1.13) | 0.98 (0.79–1.20) | 1.01 (1.01–1.01) | 1.17 (1.14–1.20) |
| LENVATINIB | 91 | 30,630 | 1.24 (1.01–1.52) | 1.13 (0.92–1.39) | 1.01 (1.01–1.01) | 1.17 (1.14–1.20) |
| PEMBROLIZUMAB | 91 | 47,669 | 0.79 (0.64–0.97) | 0.73 (0.60–0.90) | 1.01 (1.01–1.01) | 1.17 (1.13–1.20) |
| DULAGLUTIDE | 89 | 27,496 | 1.35 (1.09–1.66) | 1.27 (1.03–1.57) | 1.01 (1.01–1.01) | 1.17 (1.14–1.20) |
| SERTRALINE HYDROCHLORIDE | 86 | 35,751 | 1.00 (0.81–1.24) | 1.15 (0.93–1.42) | 1.01 (1.01–1.01) | 1.17 (1.14–1.20) |
| SODIUM OXYBATE | 85 | 57,938 | 0.61 (0.49–0.75) | 0.69 (0.56–0.85) | 1.01 (1.01–1.01) | 1.17 (1.14–1.20) |
| RIBOCICLIB | 82 | 28,841 | 1.18 (0.95–1.47) | 1.07 (0.86–1.33) | 1.01 (1.01–1.01) | 1.17 (1.14–1.20) |
| APOMORPHINE HYDROCHLORIDE | 80 | 11,420 | 2.93 (2.35–3.65) | 2.65 (2.13–3.31) | 1.01 (1.01–1.01) | 1.17 (1.14–1.20) |
| NICOTINE | 77 | 13,799 | 2.33 (1.86–2.92) | 2.36 (1.88–2.95) | 1.01 (1.01–1.01) | 1.17 (1.14–1.20) |
| CRIZOTINIB | 76 | 8,773 | 3.63 (2.90–4.55) | 3.47 (2.77–4.35) | 1.01 (1.01–1.01) | 1.17 (1.14–1.20) |
| LEVOFLOXACIN | 75 | 31,292 | 1.00 (0.79–1.25) | 0.98 (0.78–1.23) | 1.01 (1.01–1.01) | 1.17 (1.14–1.20) |
| TOFACITINIB CITRATE | 71 | 79,284 | 0.37 (0.29–0.47) | 0.34 (0.27–0.43) | 1.01 (1.01–1.01) | 1.17 (1.14–1.20) |
| DIMETHYL FUMARATE | 70 | 72,184 | 0.40 (0.32–0.51) | 0.42 (0.33–0.54) | 1.01 (1.01–1.01) | 1.17 (1.14–1.20) |
| OMALIZUMAB | 69 | 88,833 | 0.32 (0.25–0.41) | 0.34 (0.27–0.43) | 1.01 (1.01–1.01) | 1.17 (1.14–1.20) |
| TOPIRAMATE | 68 | 8,638 | 3.30 (2.60–4.19) | 3.78 (2.98–4.80) | 1.01 (1.01–1.01) | 1.16 (1.13–1.20) |
| OXALIPLATIN | 67 | 35,580 | 0.78 (0.61–0.99) | 0.75 (0.59–0.95) | 1.01 (1.01–1.01) | 1.17 (1.14–1.20) |
| EVOLOCUMAB | 66 | 58,901 | 0.46 (0.36–0.59) | 0.41 (0.32–0.52) | 1.01 (1.01–1.01) | 1.17 (1.14–1.20) |
| OFATUMUMAB | 65 | 39,894 | 0.68 (0.53–0.86) | 0.73 (0.58–0.94) | 1.01 (1.01–1.01) | 1.17 (1.14–1.20) |
| ALBUTEROL SULFATE | 64 | 8,455 | 3.17 (2.48–4.05) | 3.06 (2.39–3.91) | 1.01 (1.01–1.01) | 1.17 (1.14–1.20) |
| PEGINTERFERON ALFA-2A | 64 | 11,270 | 2.37 (1.85–3.03) | 2.51 (1.96–3.21) | 1.01 (1.01–1.01) | 1.17 (1.14–1.20) |
| CARBOPLATIN | 64 | 39,391 | 0.67 (0.53–0.86) | 0.63 (0.49–0.80) | 1.01 (1.01–1.01) | 1.17 (1.14–1.20) |
| GLATIRAMER ACETATE | 63 | 35,575 | 0.73 (0.57–0.94) | 0.79 (0.62–1.01) | 1.01 (1.01–1.01) | 1.17 (1.14–1.20) |
| ERLOTINIB HYDROCHLORIDE | 63 | 10,634 | 2.48 (1.93–3.17) | 2.11 (1.65–2.71) | 1.01 (1.01–1.01) | 1.17 (1.14–1.20) |
| POLYETHYLENE GLYCOL 3350 | 61 | 15,009 | 1.69 (1.32–2.18) | 1.56 (1.21–2.01) | 1.01 (1.01–1.01) | 1.17 (1.14–1.20) |
| SACUBITRIL\VALSARTAN | 61 | 57,135 | 0.44 (0.34–0.57) | 0.41 (0.32–0.52) | 1.01 (1.01–1.01) | 1.16 (1.13–1.19) |
| ENFORTUMAB VEDOTIN-EJFV | 61 | 2,462 | 10.56 (8.19–13.62) | 9.61 (7.45–12.40) | 1.01 (1.01–1.01) | 1.17 (1.14–1.20) |
| TERIFLUNOMIDE | 61 | 44,410 | 0.57 (0.44–0.73) | 0.57 (0.45–0.74) | 1.01 (1.01–1.01) | 1.17 (1.14–1.20) |
| LIRAGLUTIDE | 59 | 13,353 | 1.84 (1.43–2.38) | 1.81 (1.40–2.34) | 1.01 (1.01–1.01) | 1.17 (1.14–1.20) |
| DOCETAXEL | 58 | 65,005 | 0.37 (0.29–0.48) | 0.36 (0.28–0.46) | 1.01 (1.01–1.01) | 1.17 (1.14–1.20) |
| FLUOROURACIL | 57 | 12,890 | 1.84 (1.42–2.39) | 1.73 (1.33–2.25) | 1.01 (1.01–1.01) | 1.17 (1.14–1.20) |
| VEDOLIZUMAB | 56 | 116,646 | 0.20 (0.15–0.26) | 0.21 (0.16–0.27) | 1.01 (1.01–1.01) | 1.16 (1.13–1.20) |
| IBRUTINIB | 56 | 59,422 | 0.39 (0.30–0.51) | 0.34 (0.26–0.44) | 1.01 (1.01–1.01) | 1.16 (1.13–1.19) |
| IBUPROFEN | 55 | 28,649 | 0.80 (0.61–1.04) | 0.90 (0.69–1.18) | 1.01 (1.01–1.01) | 1.17 (1.14–1.20) |
| AMLODIPINE BESYLATE | 55 | 18,602 | 1.23 (0.94–1.60) | 1.12 (0.86–1.46) | 1.01 (1.01–1.01) | 1.17 (1.14–1.20) |
| CERTOLIZUMAB PEGOL | 55 | 63,389 | 0.36 (0.28–0.47) | 0.37 (0.28–0.48) | 1.01 (1.01–1.01) | 1.17 (1.14–1.20) |
| ABEMACICLIB | 55 | 9,578 | 2.40 (1.84–3.13) | 2.12 (1.62–2.76) | 1.01 (1.01–1.01) | 1.16 (1.13–1.20) |
| ABALOPARATIDE | 54 | 19,823 | 1.13 (0.87–1.48) | 0.96 (0.73–1.25) | 1.01 (1.01–1.01) | 1.17 (1.14–1.20) |
| AFATINIB | 54 | 11,265 | 2.00 (1.53–2.61) | 1.76 (1.35–2.30) | 1.01 (1.01–1.01) | 1.17 (1.14–1.20) |
| DENOSUMAB | 53 | 45,600 | 0.48 (0.37–0.63) | 0.39 (0.30–0.52) | 1.01 (1.01–1.01) | 1.17 (1.14–1.20) |
| MACITENTAN | 53 | 83,003 | 0.26 (0.20–0.34) | 0.24 (0.18–0.32) | 1.01 (1.01–1.01) | 1.17 (1.14–1.20) |
| LEUPROLIDE ACETATE | 53 | 63,349 | 0.35 (0.26–0.45) | 0.33 (0.25–0.43) | 1.01 (1.01–1.01) | 1.16 (1.13–1.19) |
| ZOLEDRONIC ACID | 53 | 29,369 | 0.75 (0.57–0.98) | 0.65 (0.50–0.85) | 1.01 (1.01–1.01) | 1.17 (1.14–1.20) |
| VARENICLINE TARTRATE | 52 | 9,405 | 2.31 (1.76–3.03) | 2.36 (1.80–3.10) | 1.01 (1.01–1.01) | 1.17 (1.14–1.20) |
| CARBIDOPA\LEVODOPA | 52 | 70,346 | 0.31 (0.23–0.40) | 0.26 (0.20–0.35) | 1.01 (1.01–1.01) | 1.16 (1.13–1.19) |
| TRASTUZUMAB | 50 | 20,042 | 1.04 (0.79–1.37) | 0.98 (0.74–1.29) | 1.01 (1.01–1.01) | 1.17 (1.14–1.20) |
| AZELASTINE HYDROCHLORIDE\FLUTICASONE PROPIONATE | 47 | 1,010 | 20.27 (15.12–27.18) | 20.20 (15.06–27.09) | 1.01 (1.01–1.01) | 1.17 (1.14–1.20) |
| OCTREOTIDE ACETATE | 44 | 57,214 | 0.32 (0.24–0.43) | 0.30 (0.22–0.40) | 1.01 (1.01–1.01) | 1.17 (1.14–1.20) |
| CHLORHEXIDINE GLUCONATE | 44 | 1,363 | 13.86 (10.26–18.71) | 13.95 (10.32–18.84) | 1.01 (1.01–1.01) | 1.17 (1.14–1.20) |
| RIVAROXABAN | 44 | 88,912 | 0.20 (0.15–0.27) | 0.18 (0.13–0.24) | 1.01 (1.01–1.01) | 1.16 (1.13–1.19) |
| DICHLORPHENAMIDE | 44 | 2,434 | 7.65 (5.67–10.30) | 8.47 (6.29–11.42) | 1.01 (1.01–1.01) | 1.17 (1.14–1.20) |
| BEVACIZUMAB | 44 | 38,174 | 0.48 (0.36–0.64) | 0.45 (0.34–0.61) | 1.01 (1.01–1.01) | 1.17 (1.14–1.20) |
| MARIBAVIR | 43 | 695 | 27.39 (20.11–37.31) | 27.83 (20.43–37.92) | 1.01 (1.01–1.01) | 1.17 (1.14–1.20) |
| IMATINIB MESYLATE | 43 | 17,685 | 1.01 (0.75–1.36) | 1.00 (0.74–1.35) | 1.01 (1.01–1.01) | 1.17 (1.14–1.20) |
| NALTREXONE | 42 | 21,644 | 0.81 (0.60–1.09) | 0.98 (0.72–1.32) | 1.01 (1.01–1.01) | 1.17 (1.14–1.20) |
| TIRZEPATIDE | 42 | 22,333 | 0.78 (0.58–1.06) | 0.79 (0.58–1.07) | 1.01 (1.01–1.01) | 1.17 (1.14–1.20) |
| CETYLPYRIDINIUM CHLORIDE | 42 | 266 | 77.88 (56.00–108.31) | 87.01 (62.51–121.11) | 1.01 (1.01–1.01) | 1.17 (1.14–1.20) |
| FLUTICASONE FUROATE\VILANTEROL TRIFENATATE | 42 | 9,555 | 1.83 (1.35–2.48) | 1.61 (1.19–2.18) | 1.01 (1.01–1.01) | 1.17 (1.14–1.20) |
| ALPELISIB | 40 | 6,477 | 2.58 (1.89–3.52) | 2.36 (1.73–3.23) | 1.01 (1.01–1.01) | 1.16 (1.13–1.20) |
| ESKETAMINE | 40 | 7,847 | 2.13 (1.56–2.90) | 2.33 (1.71–3.18) | 1.01 (1.01–1.01) | 1.17 (1.14–1.20) |
| UMECLIDINIUM BROMIDE\VILANTEROL TRIFENATATE | 40 | 5,678 | 2.95 (2.16–4.02) | 2.49 (1.83–3.40) | 1.01 (1.01–1.01) | 1.17 (1.14–1.20) |
| LETROZOLE | 40 | 18,121 | 0.92 (0.67–1.25) | 0.79 (0.58–1.08) | 1.01 (1.01–1.01) | 1.17 (1.14–1.20) |
| APIXABAN | 39 | 46,888 | 0.34 (0.25–0.47) | 0.29 (0.21–0.39) | 1.01 (1.01–1.01) | 1.16 (1.13–1.20) |
| OSELTAMIVIR PHOSPHATE | 39 | 7,196 | 2.26 (1.65–3.10) | 2.68 (1.95–3.67) | 1.01 (1.01–1.01) | 1.17 (1.14–1.20) |
| ENFORTUMAB VEDOTIN | 39 | 2,022 | 8.17 (5.95–11.21) | 7.46 (5.43–10.24) | 1.01 (1.01–1.01) | 1.17 (1.14–1.20) |
| DULOXETINE HYDROCHLORIDE | 39 | 21,141 | 0.77 (0.56–1.05) | 0.77 (0.57–1.06) | 1.01 (1.01–1.01) | 1.17 (1.14–1.20) |
| ENTRECTINIB | 38 | 1,290 | 12.60 (9.12–17.41) | 12.02 (8.70–16.61) | 1.01 (1.01–1.01) | 1.17 (1.14–1.20) |
| CYCLOSPORINE | 38 | 14,852 | 1.06 (0.77–1.46) | 1.03 (0.75–1.41) | 1.01 (1.01–1.01) | 1.17 (1.14–1.20) |
| FLUTICASONE PROPIONATE\SALMETEROL XINAFOATE | 38 | 9,504 | 1.67 (1.21–2.29) | 1.49 (1.08–2.05) | 1.01 (1.01–1.01) | 1.17 (1.14–1.20) |
| SORAFENIB | 38 | 18,606 | 0.85 (0.62–1.17) | 0.82 (0.59–1.12) | 1.01 (1.01–1.01) | 1.17 (1.14–1.20) |
| MAGNESIUM SULFATE\POTASSIUM SULFATE\SODIUM SULFATE | 38 | 6,830 | 2.32 (1.69–3.19) | 2.12 (1.54–2.92) | 1.01 (1.01–1.01) | 1.17 (1.14–1.20) |
| REGORAFENIB | 37 | 18,873 | 0.81 (0.59–1.12) | 0.78 (0.57–1.08) | 1.01 (1.01–1.01) | 1.17 (1.14–1.20) |
| APALUTAMIDE | 37 | 4,755 | 3.26 (2.36–4.50) | 2.90 (2.09–4.01) | 1.01 (1.01–1.01) | 1.17 (1.14–1.20) |
| BUDESONIDE | 37 | 8,467 | 1.82 (1.32–2.52) | 1.91 (1.38–2.63) | 1.01 (1.01–1.01) | 1.17 (1.14–1.20) |
| AXITINIB | 36 | 10,625 | 1.41 (1.02–1.96) | 1.34 (0.96–1.86) | 1.01 (1.01–1.01) | 1.17 (1.14–1.20) |
| ERENUMAB-AOOE | 36 | 28,475 | 0.52 (0.38–0.73) | 0.54 (0.39–0.75) | 1.01 (1.01–1.01) | 1.17 (1.14–1.20) |
| LEVOTHYROXINE SODIUM | 36 | 41,393 | 0.36 (0.26–0.50) | 0.35 (0.25–0.49) | 1.01 (1.01–1.01) | 1.17 (1.14–1.20) |
| VEMURAFENIB | 36 | 6,486 | 2.32 (1.67–3.21) | 2.29 (1.65–3.18) | 1.01 (1.01–1.01) | 1.17 (1.14–1.20) |
| OXYMETAZOLINE HYDROCHLORIDE | 35 | 2,303 | 6.41 (4.59–8.95) | 6.30 (4.51–8.80) | 1.01 (1.01–1.01) | 1.17 (1.13–1.20) |
| AZITHROMYCIN ANHYDROUS | 35 | 5,882 | 2.48 (1.78–3.46) | 2.56 (1.84–3.58) | 1.01 (1.01–1.01) | 1.17 (1.14–1.20) |
| ONABOTULINUMTOXINA | 35 | 25,362 | 0.57 (0.41–0.80) | 0.59 (0.42–0.82) | 1.01 (1.01–1.01) | 1.17 (1.14–1.20) |
| FLUTICASONE FUROATE\UMECLIDINIUM BROMIDE\VILANTEROL TRIFENATATE | 34 | 5,041 | 2.82 (2.01–3.95) | 2.38 (1.70–3.34) | 1.01 (1.01–1.01) | 1.17 (1.14–1.20) |
| DICLOFENAC SODIUM | 33 | 16,089 | 0.85 (0.61–1.20) | 0.76 (0.54–1.07) | 1.01 (1.01–1.01) | 1.17 (1.14–1.20) |
| TIPIRACIL HYDROCHLORIDE\TRIFLURIDINE | 33 | 8,413 | 1.63 (1.16–2.30) | 1.53 (1.08–2.15) | 1.01 (1.01–1.01) | 1.17 (1.14–1.20) |
| PANITUMUMAB | 33 | 7,558 | 1.82 (1.29–2.56) | 1.74 (1.24–2.45) | 1.01 (1.01–1.01) | 1.17 (1.14–1.20) |
| DASABUVIR\OMBITASVIR\PARITAPREVIR\RITONAVIR | 32 | 14,486 | 0.92 (0.65–1.30) | 0.93 (0.66–1.32) | 1.01 (1.01–1.01) | 1.17 (1.14–1.20) |
| FLUTICASONE FUROATE | 32 | 2,156 | 6.25 (4.41–8.87) | 5.98 (4.21–8.48) | 1.01 (1.01–1.01) | 1.17 (1.14–1.20) |
| NATALIZUMAB | 32 | 59,052 | 0.22 (0.16–0.32) | 0.24 (0.17–0.34) | 1.01 (1.01–1.01) | 1.17 (1.14–1.20) |
| PHENTERMINE HYDROCHLORIDE\TOPIRAMATE | 32 | 1,909 | 7.08 (4.99–10.04) | 7.41 (5.22–10.51) | 1.01 (1.01–1.01) | 1.17 (1.13–1.20) |
| LEVONORGESTREL | 31 | 127,827 | 0.10 (0.07–0.14) | 0.12 (0.09–0.18) | 1.01 (1.01–1.01) | 1.18 (1.15–1.21) |
| LINEZOLID | 31 | 8,936 | 1.44 (1.01–2.06) | 1.40 (0.98–1.99) | 1.01 (1.01–1.01) | 1.17 (1.14–1.20) |
| METFORMIN HYDROCHLORIDE | 31 | 17,204 | 0.75 (0.53–1.06) | 0.69 (0.49–0.99) | 1.01 (1.01–1.01) | 1.17 (1.14–1.20) |
| CLOPIDOGREL BISULFATE | 30 | 18,165 | 0.69 (0.48–0.98) | 0.60 (0.42–0.86) | 1.01 (1.01–1.01) | 1.17 (1.14–1.20) |
| CORTICOTROPIN | 30 | 11,434 | 1.09 (0.76–1.56) | 1.08 (0.75–1.54) | 1.01 (1.01–1.01) | 1.17 (1.14–1.20) |
| ABIRATERONE ACETATE | 30 | 13,908 | 0.90 (0.63–1.28) | 0.80 (0.56–1.15) | 1.01 (1.01–1.01) | 1.17 (1.13–1.20) |
| ATORVASTATIN | 30 | 18,311 | 0.68 (0.48–0.97) | 0.62 (0.44–0.89) | 1.01 (1.01–1.01) | 1.17 (1.14–1.20) |
| TIOTROPIUM BROMIDE MONOHYDRATE | 30 | 10,469 | 1.19 (0.83–1.71) | 1.02 (0.72–1.47) | 1.01 (1.01–1.01) | 1.17 (1.14–1.20) |
| RITUXIMAB | 30 | 50,691 | 0.24 (0.17–0.35) | 0.24 (0.17–0.34) | 1.01 (1.01–1.01) | 1.17 (1.14–1.20) |
| DABRAFENIB MESYLATE | 30 | 12,596 | 0.99 (0.69–1.42) | 0.96 (0.67–1.37) | 1.01 (1.01–1.01) | 1.17 (1.14–1.20) |

ROR, reporting odds ratio; CI, confidence interval
